# A green leaf volatile, (*Z*)-3-hexenyl-acetate, mediates differential oviposition by *Spodoptera frugiperda* on maize and rice

**DOI:** 10.1186/s12915-023-01642-x

**Published:** 2023-06-19

**Authors:** Jiali Wang, Jiaqi Wei, Ting Yi, Ya-Ya Li, Tian Xu, Li Chen, Hanhong Xu

**Affiliations:** 1 National Key Laboratory of Green Pesticide, South China Agricultural University, Guangzhou, Guangdong China; 2 School of Life Sciences/Hebei Basic Science Center for Biotic Interaction, Institute of Life Science and Green Development, Hebei University, Baoding, 071002 China; 3 College of Forestry, Nanjing Forestry University, Nanjing, China

**Keywords:** *Spodoptera frugiperda*, Volatile organic compounds, Oviposition preference, Oviposition stimulation, Olfactory receptor, RNA interference

## Abstract

**Background:**

Insects rely on chemosensory perception, mainly olfaction, for the location of mates, food sources, and oviposition sites. Plant-released volatile compounds guide herbivorous insects to search for and locate their host plants, further helping them to identify suitable positions for oviposition. The fall armyworm *Spodoptera frugiperda* (*S. frugiperda*) was found to invade China in 2019 and has since seriously threatened multiple crops, particularly maize and rice. However, the chemical and molecular mechanisms underlying oviposition preference in this pest are not fully understood. Here, the oviposition preference of *S. frugiperda* on maize and rice plants was investigated.

**Results:**

GC-EAD and GC–MS/MS techniques were used to identify the antennally active volatiles from maize and rice plants. The attraction and oviposition stimulation of identified components to female adults were tested in both laboratory and field settings. The odorant receptors (ORs) on female antennae were expressed in *Xenopus oocytes*, and their functions evaluated by RNAi. Ten and eleven compounds of maize and rice plants, respectively, were identified to possess electrophysiological activity from headspace volatiles. Among these compounds, (*Z*)-3-hexenyl-acetate specifically presented in maize volatiles was found to play a critical role in attracting females and stimulating oviposition compared to rice volatiles. Among the cloned ORs on the antennae of both sexes, *SfruOR23* with highly female-biased expression mediated the responses of females to (*Z*)-3-hexenyl-acetate. Knockdown of *SfruOR23* using RNAi markedly reduced the electrophysiological response of female antennae and oviposition preference to the compound.

**Conclusions:**

(*Z*)-3-Hexenyl-acetate is a key volatile mediating the host and oviposition preference of *S. frugiperda* on maize. The olfactory receptor of (*Z*)-3-hexenyl-acetate was identified to be *SfruOR23*, which is mainly expressed in the antennae of *S. frugiperda*.

**Supplementary Information:**

The online version contains supplementary material available at 10.1186/s12915-023-01642-x.

## Background

Herbivorous insects rely on volatile chemical cues from host plants to find suitable habitats, including the selection of host plants for feeding and reproduction. These cues can be general green leaf volatiles, host plant-specific volatile organic compounds (VOCs), or a specific blend of VOCs unique to a plant species [1, 2]. Gravid female insects strongly rely on these chemical cues to locate the suitable host plant for oviposition [3, 4]. As an initial step prior to oviposition, these host plant-derived VOCs guide adult females to the vicinity of the oviposition site. For example, nonanal and decanal emitted by maize seedlings attract the gravid female *Ostrinia nubilalis* [5]. Once they approach an oviposition site, furthermore, gravid females rely on the presence of some key VOCs to make oviposition decisions, which can be general green leaf volatiles and/or host-specific VOCs [6]. For example, ethyl (*E*, *Z*)-2,4-decadienoate from pear increases the number of eggs deposited by *Cydia pomonella* in no-choice assays [7]. Moreover, some host-plant volatiles have a dual function in attracting gravid females and stimulating their oviposition behavior. For instance, γ-octalactone is a critical VOC from mango that can be sensed by gravid female *Bactrocera dorsalis* for host selection [8] and oviposition stimulation [9]*.*

Oligophagous or monophagous insects have highly specialized chemosensory systems, enabling them to recognize specific compounds or complex mixtures to select their hosts or oviposit [10, 11]. On the other hand, polyphagous insects have a wide range of acceptable host species and exhibit a hierarchy of oviposition preferences among the host species. A mated female adult usually lays most of her eggs on a preferred plant species [12–14], while fewer eggs will be laid or the oviposition will be delayed when only less preferred host species are presented [15, 16]. The maternal oviposition choices of herbivorous insects should be adaptive to maximize the fitness of their offspring [17]. In most studies on oviposition selection, insects are allowed to choose freely between two plants for oviposition [18]. Though this design allows the confirmation of oviposition preference, it is difficult to determine the factors that cause the phenomenon [19].

The fall armyworm (FAW), *Spodoptera frugiperda* (*S. frugiperda*, Lepidoptera: Noctuidae), is a highly invasive pest with destructive impacts [20–23]. Originally located in tropical and subtropical America, FAW first invaded Africa in 2016 and rapidly spread across sub-Saharan Africa by 2017 [21], posing significant threats to food security [24–26]. It subsequently invaded Southeast Asia, then spreaded from Myanmar to Yunnan Province in southern China in December 2018 [27–31]. Since then, *S. frugiperda* has spread across 26 provinces of China and caused damage to twenty-one crops of seven families (Gramineae, Cruciferae, Solanaceae, Ginger, Leguminosae, Platanaceae, and Taro) and eight types of grass [32]. There are two distinct host strains of FAW, “corn strain” and “rice strain” [33–35], that resulted from reproductive isolation or selective effects of different hosts [33–36]. The populations of FAW in China (Guangdong, Yunnan, Beijing, etc.) were determined to be “a hybrid of corn strain and rice strain” (corn strain male × rice strain female) according to two molecular markers, cytochrome oxidase subunit1 (*CO I*) and triosephosphate isomerase (*Tpi*) [37–39]. It mainly damages maize and has an apparent preference to oviposit on maize plants rather than on rice plants [40], while the rice-fed larvae exhibit a lower survival rate than maize-fed larvae. In addition, it has been observed that different varieties of maize lead to differences in oviposition attraction, which can be due to the different types of volatiles being produced [41]. Previous researches have been focused on the analysis of VOCs used by *S. frugiperda* to locate suitable maize hosts, majorly by comparing the volatile profiles of different maize varieties [41, 42]. Some specific compounds such as (*E*)-*α*-bergamotene and methyl salicylate have been found to attract female *S. frugiperda* and promote oviposition, while (*E*)-4,8-dimethyl-1,3,7-nonatriene has been found to decrease the egg masses [41]. Several other active compounds have also been identified with electroantennography (EAG) [43].

Insects typically rely on their olfactory system to discriminate host plant VOCs and make oviposition decisions [44–46]. The main organ that is responsible for detecting odors in insects is the antennae. Odorants are primarily detected by odorant receptors (OR) located on the dendritic membrane of olfactory sensory neurons (OSN) in the antennae. Upon the activation by odor ligands, ORs are able to produce olfactory signals, which are then transmitted to the central nervous system and later trigger corresponding behavioral responses [47–51] such as approaching the particular odor source or selecting a specific host plant for oviposition [47, 52]. For example, *DmOR19a* modulates the selective oviposition behavior, and *DmOR7a* stimulates the oviposition behavior of *Drosophila* females [6, 53]; *Helicoverpa assulta OR67* is mainly responsible for locating the tobacco plants and oviposition sites [54]. Previously, a total of 82 OR genes of the chemoreception-related gene family have been identified in *S. frugiperda* at the genomic level [55]. Six candidate pheromone receptors (PRs) were identified in the FAW [56], with *SfruOR13* and *SfruOR16* responding to the sex pheromone components Z9-14:OAc and Z9-12:OAc [57]. However, the ORs responsible for detecting plant volatiles in *S. frugiperda* have not yet been identified. Clarifying the peripheral neurosensory mechanisms of these volatile compounds is crucial for the development of insect behavioral modulators that target olfactory receptors. In this study, we aim to identify (1) the causes of oviposition preference by corn-strain of *S. frugiperda* on maize plants over rice plants, (2) the key volatile compound(s) responsible for the oviposition preference on maize plants, (3) the receptors that are sensitive to the specific oviposition stimulant.

## Results

### Two-choice oviposition preference assay on host plant

**Fig. 1:**
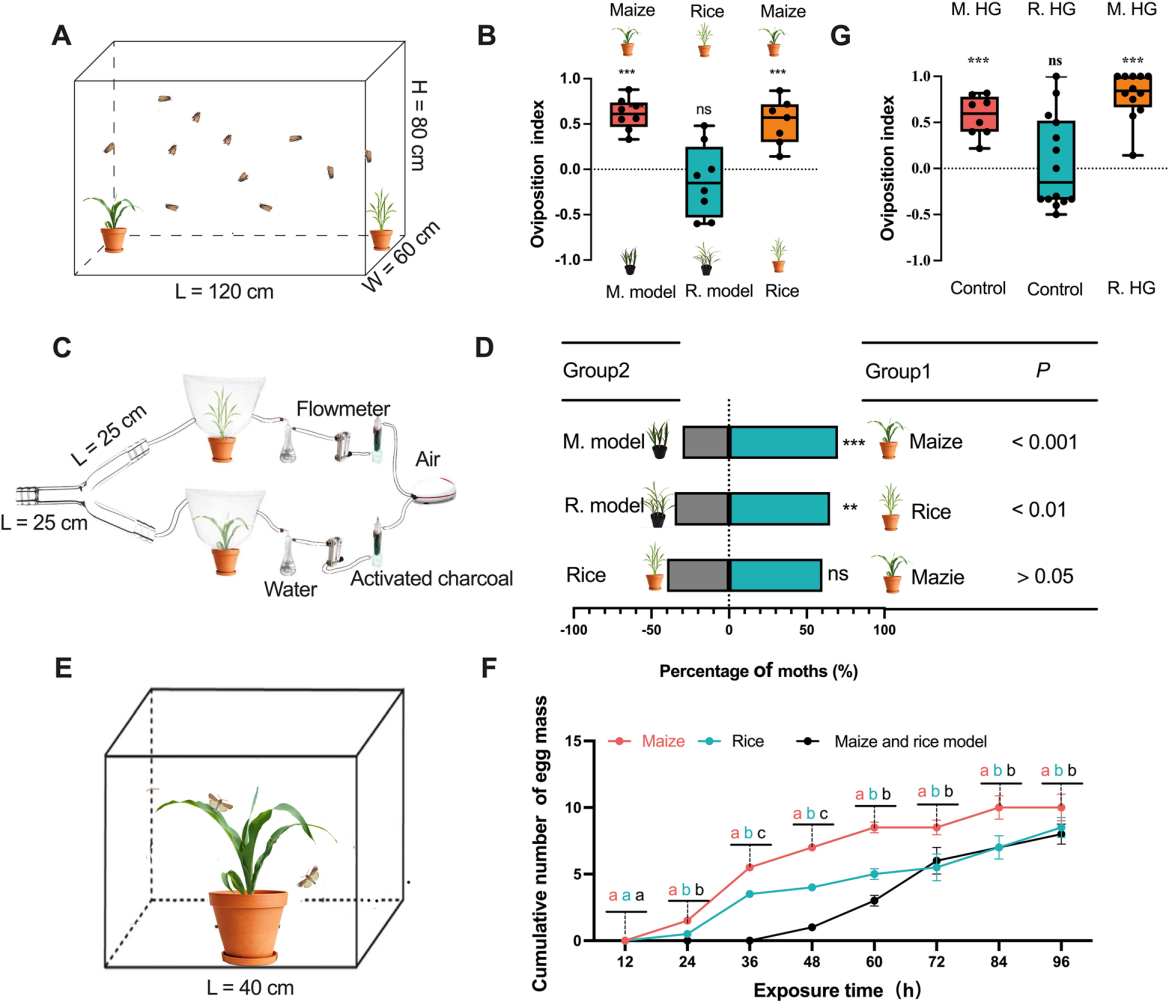
Oviposition and olfactory preferences of female *S. frugiperda. ***A** Schematic illustration of two-choice oviposition assay. **B** Oviposition indexes of maize/maize model, rice/rice model, and maize/rice. (Chi-square test, *n* = 10; *** P < 0.001). Oviposition index = (number of egg masses on maize or rice − number of egg masses on model plant)/(number of egg masses on maize or rice + number of egg masses on model plant). M. model: maize model; R. model: rice model. **C** Schematic illustration of two-choice Y-maze assay. **D** Behavioral responses of female *S. frugiperda* adults to maize plant vs. maize model, rice plant vs. rice model, and maize plant vs. rice plant (Chi-square test, *n* = 30; ns, *P* > 0.05; *, *P* < 0.05; **,* P* < 0.01; ***,* P* < 0.001). M. model: Maize model; R. model: Rice model. **E** Schematic illustration of no-choice oviposition stimulating experiment. **F** The cumulative numbers (mean ± SE) of egg masses deposited by females from 24 to 96 h on maize/rice/model plants were analyzed by one-way ANOVA followed by Tukey’s pairwise test, *n* = 30; *P* < 0.05. **G** Oviposition indexes of maize homogenate on maize model/control (water on maize model), rice homogenate on rice model/control (water on rice model), and maize homogenate/rice homogenate. (Chi-square test, *n* = 10; *** P < 0.001). M. HG: Maize homogenate on maize model; R. HG: Rice homogenate on rice model; M. model: Maize model; R. model: rice model

Based on the analysis of two molecular markers (*CO I* and* Tpi*) in 255 samples from 17 sites (Table S1), we confirmed that the population of *S. frugiperda* that invaded China was a heterozygous corn strain resulting from corn strain male mated to rice strain female. The oviposition preference of female *S. frugiperda* adults toward maize plants was identified in a two-choice assay. Results showed that compared to the model of maize, (*P* < 0.001). However, no significant preference in oviposition was shown between the rice plants and the rice models seedings (*P* > 0.05). Besides, clear oviposition preference of female *S. frugiperda* adults was observed toward maize plants compared with rice plants, with significantly more egg masses deposited on the maize plants (*P* < 0.001) (Fig. 1B).

### Two-choice olfactory preference assay on host plant

Compared to the maize or rice model seedings, female *S. frugiperda* adults showed an olfactory preference for maize or rice plants (*P* < 0.001) while not showing a difference in olfaction choices between maize and rice (*P* > 0.05) in the two-choice olfactory assays (Fig. 1D). Thus, the oviposition preference of female *S. frugiperda* adults between maize and rice cannot be attributed to the olfactory preference.

### No-choice oviposition stimulation assay on host plant

The oviposition-stimulation effect of maize and rice on female *S. frugiperda* was then tested (Fig. 1E)*.* The numbers of egg masses deposited by female exposed to maize for 24 h to 96 h (*P* < 0.01) were significantly greater than that exposed to rice and blank control, whereas the latter two treatments showed no significant difference in results from 60 to 96 h (*P* > 0.05). Thus, the oviposition preference between maize and rice was caused by the oviposition stimulation (Fig. 1F).

### Two-choice oviposition preference assay on homogenate

A two-choice assay was conducted to identify the oviposition preference of female *S. frugiperda* adults towards maize and rice homogenates. Compared to rice homogenate and the control experiment with water treated on maize model seedings, female *S. frugiperda* adults showed a clear preference for maize homogenate with significantly more egg masses deposited on maize homogenate (*P* < 0.001) (Fig. 1G). Meanwhile, no difference was shown in oviposition between the rice homogenate and the control group (*P* > 0.05). Additionally, *S. frugiperda* showed a significant preference for the odors of both maize and rice homogenate compared to the control group. However, there was no significant preference observed between the odors of maize and rice plant (*P* > 0.05) (Fig. S1).

### Antennally-active volatile released by maize and rice

**Fig. 2:**
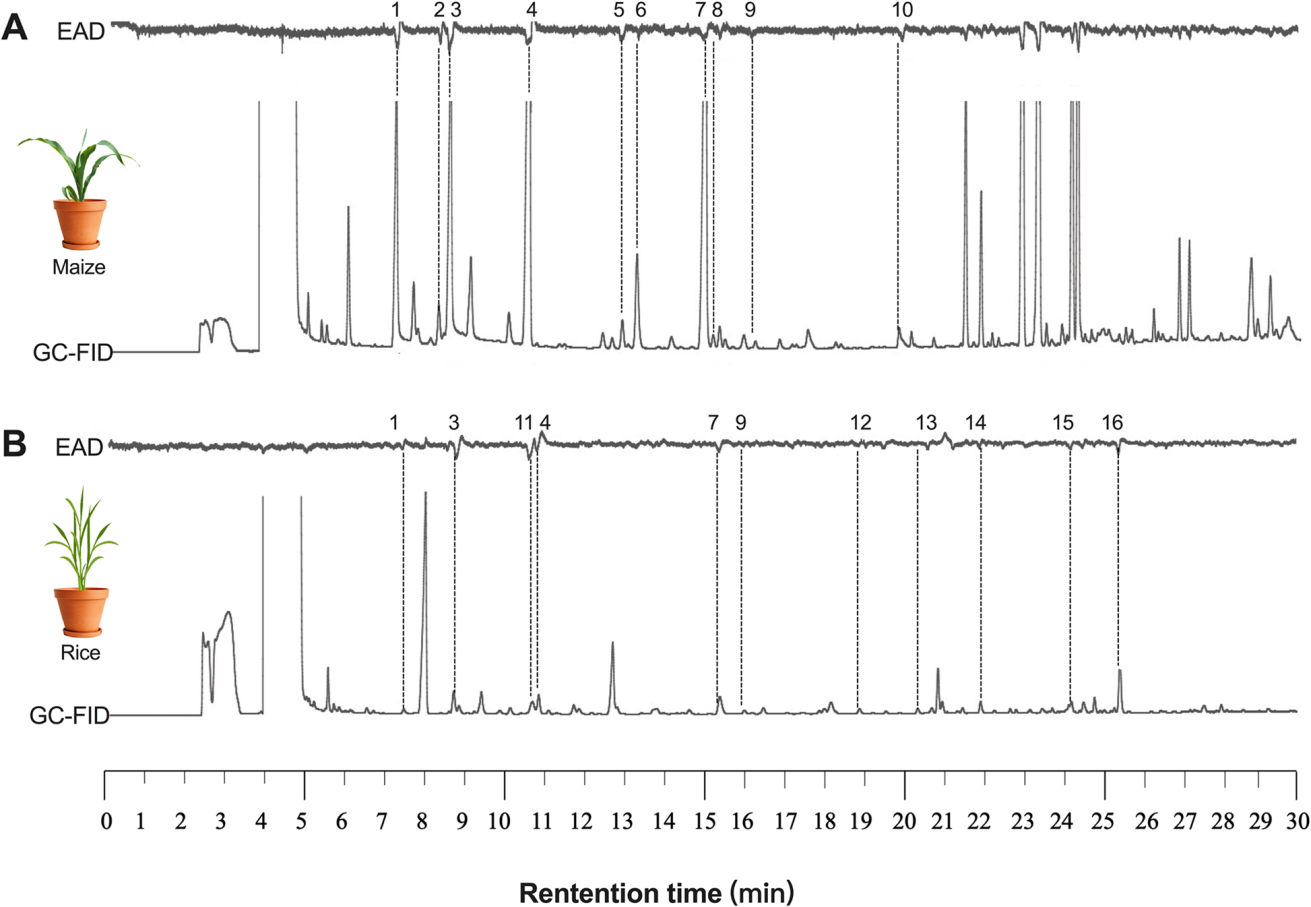
GC-EAD responses of female *S. frugiperda* to headspace volatile samples of maize (**A**) and rice (**B**). The antennally-active compounds identified are listed in Table 1. In the GC-EAD analysis of maize, the large peaks with retention times later than 21 min that trigger significant EAD responses are typical impurities derived from volatile collection tubing

The amounts and types of VOCs were various for different strains of plants[41]. The specific volatiles of maize and rice are specific only to the specific strains used in this study. Ten and eleven volatile compounds, respectively produced by maize and rice, were observed to elicit antennal responses of female *S. frugiperda* consistently (Fig. 2). The identities of the responsible compounds are summarized in Table 1. Specifically, (*E*)-2-pentenal, (*Z*)*-3*-hexenyl-acetate, (*Z*)-2-pentenol, nonanal, and *β*-caryophyllene were only found in maize VOCs, whereas 2-methy-1-buntanol, linalool, undecanal, methyl benzoate, benzyl alcohol, and pentadecanal exclusively exist in rice VOCs. Hexanal, (*Z*)*-*3-hexanal, (*Z*)*-*3-hexenal, (*E*)-2-hexenal, (*Z*)-3-hexenol and (*Z*)-2-hexenol were common to both host plants.

**Table 1:** Antennally-active compounds released from maize and rice

Peak No	Compound	Retention time (min)	Maize	Rice
1	Hexanal	7.38	+	+
2	(*E*)-2-Pentenal	8.33	+	−
3	(*Z*)-3-Hexenal	8.61	+	+
4	(*E*)-2-Hexenal	10.960	+	+
5	(*Z*)-3-Hexenyl-acetate	12.87	+	−
6	(*Z*)-2-Pentenol	13.48	+	−
7	(*Z*)-3-Hexenol	15.32	+	+
8	Nonanal	15.79	+	−
9	(*Z*)-2-Hexenol	15.95	+	+
10	*β*-Caryophyllene	19.87	+	−
11	2-Methy-1-butanol	10.68	−	+
12	Linalool	18.85	−	+
13	Undecanal	20.43	−	+
14	Methyl benzoate	21.97	−	+
15	Benzyl alcohol	24.28	−	+
16	Pentadecanal	25.45	−	+

“ + ” means detection of the volatile component from the tested plant

“ − ” means no detection of the volatile component from the tested plant

### Oviposition preference on antennally-active host volatiles

**Fig. 3:**
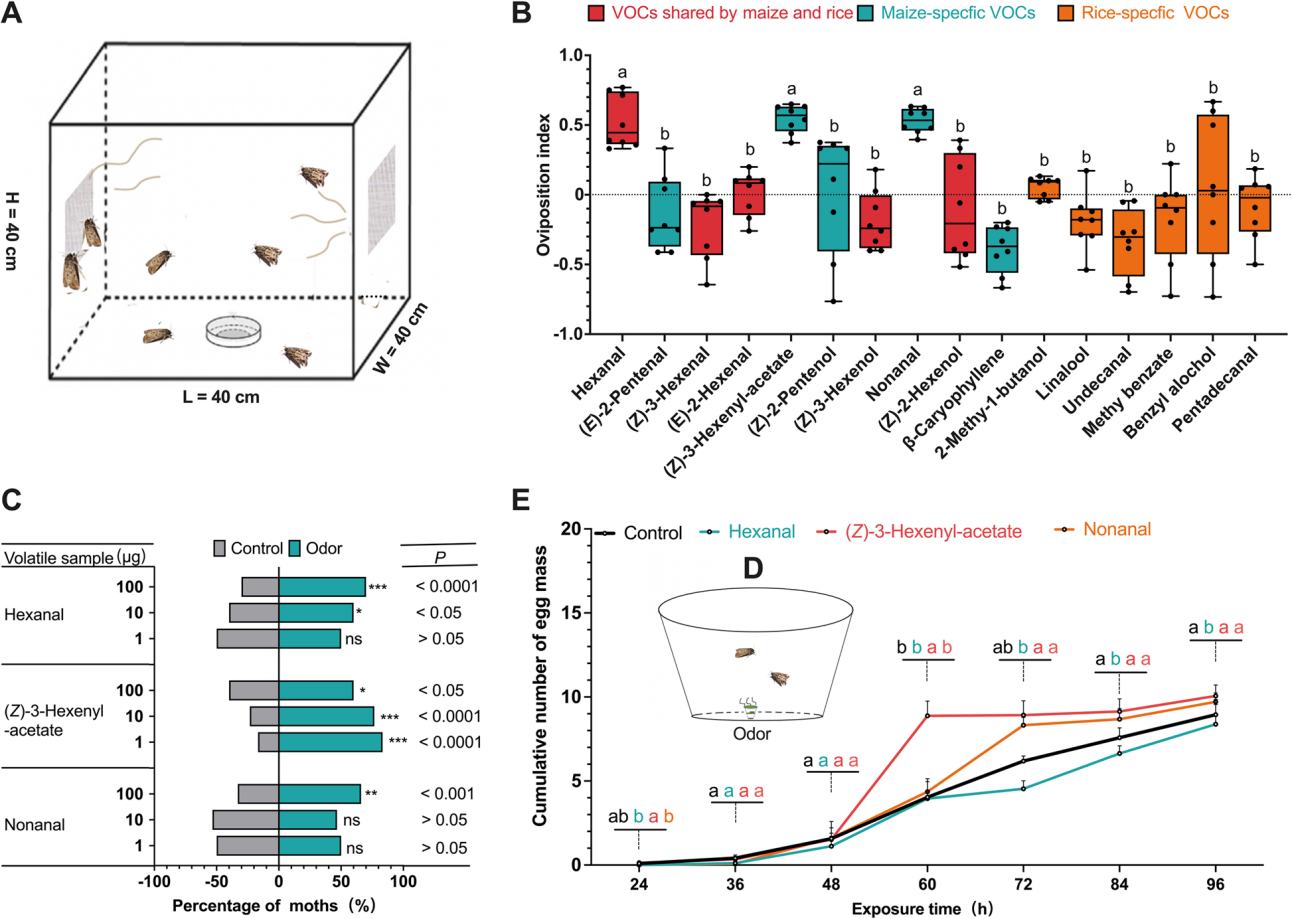
Oviposition and olfactory preference to antennally-active compounds from maize and rice. **A** Schematic illustration of two-choice oviposition assay. **B** Oviposition indexes of females toward 16 VOCs. Different letters above the bars indicate a significant difference (one-way ANOVA followed by Tukey’s pairwise test, *n* = 8, *P* < 0.05). **C** Behavioral responses of female *S. frugiperda* adults to the three active volatile compounds at 1–100 μg (chi-square test, *n* = 30; ns, *P* > 0.05; *: *P* < 0.05; **:* P* < 0.01; ***:* P* < 0.001). **D** Schematic illustration of no-choice oviposition stimulating experiment. **E** The cumulative numbers (mean ± SE) of egg mass deposited by females from 24 to 96 h. Analyzed by one-way ANOVA followed by Tukey’s pairwise test, *n* = 30; *P* < 0.05

To investigate the influence of 16 antennally-active VOCs from maize and rice on *S. frugiperda* oviposition, two-choice oviposition assays were conducted in cages (Fig. 3A). Three compounds from maize, namely hexanal, (*Z*)-3-hexenyl-acetate, and nonanal, were significantly preferred compared to other odors at the doses of 10 and 100 μg (*P* < 0.05) (Fig. 3B, Fig. S2). Maize-specific (*E*)-2-pentenal, *β*-caryophyllene, rice-specific linalool, methyl benzoate, and pentadecanal, as well as (*Z*)-3-hexanal and (*Z*)-3-hexenol in common for maize and rice turned out to be aversive for *S. frugiperda* at 100 μg (Fig. S2).

### Y-maze assay olfaction preference on antennally-active host volatiles

The attractiveness of the three oviposition-preferred compounds was further investigated in Y-maze experiments. When tested against the control (*n*-hexane), female moths showed preferences for hexanal (10 μg:* P* < 0.05; 100 μg: *P* < 0.01), (*Z*)-3-hexenyl-acetate (1 μg:* P* < 0.5; 10 μg:* P* < 0.001; 100 μg:* P* < 0.001), and nonanal (100 μg: *P* < 0.05). The preferences for low doses of hexanal (1 μg) and nonanal (1 and 10 μg) were not significant (Fig. 3C).

### Oviposition stimulation assays on oviposition preference-active host volatiles

Whether the three oviposition-preferring compounds stimulated oviposition of female *S. frugiperda* were then tested (Fig. 3D)*.* The number of egg masses deposited by females exposed to (*Z*)*-*3-hexenyl-acetate was significantly greater than that in the control (n-hexane) at 60 h (*P* < 0.05), while the difference tended to disappear with longer observation time (72 h, *P* > 0.05; 84 h,* P* > 0.05; 96 h:* P* > 0.05). There was no significant difference among hexanal, nonanal, and control across all periods. Thus, (*Z*)-3-hexenyl-acetate was able to stimulate the oviposition of female *S. frugiperda* (Fig. 3E).

### Effect of (*Z*)-3-hexenyl-acetate on oviposition preference

**Fig. 4:**
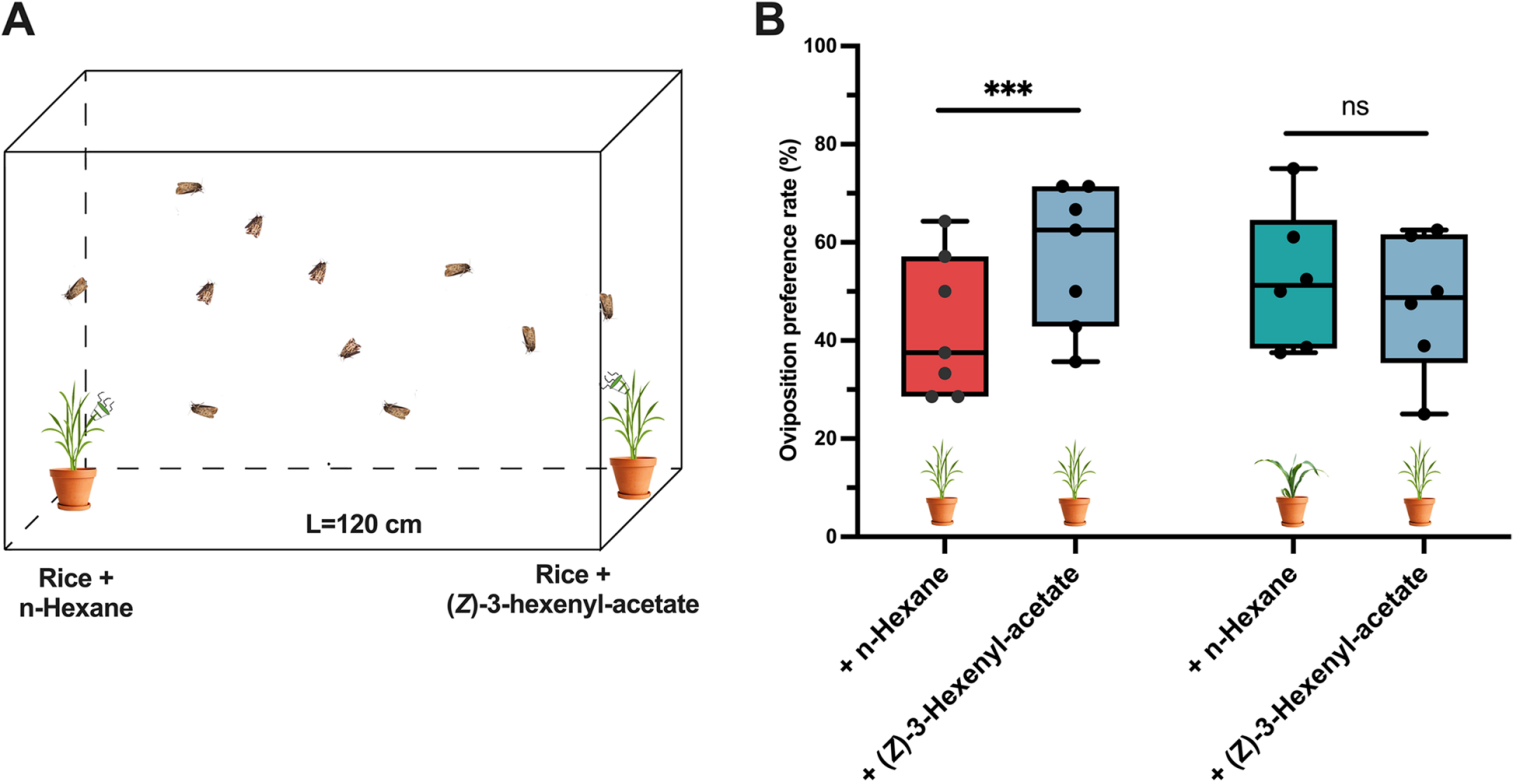
Influence of (*Z*)-3-hexenyl-acetate on *S. frugiperda* oviposition preference. **A** Schematic illustration of cage oviposition assay with (*Z*)-3-hexenyl-acetate. **B** Oviposition rates of females from choice tests between (*Z*)-3-hexenyl-acetate exposed rice and rice or (*Z*)-3-hexenyl-acetate exposed rice and maize. Oviposition preference rate = number of egg masses on maize or rice + (*Z*)-3-hexenyl-acetate/(total number of egg masses on rice or maize + (*Z*)-3-hexenyl-acetate and rice/maize). (Chi-square test, *n* = 8; ns: *P* > 0.05; ***:* P* < 0.001)

Based on the above results, the role of (*Z*)-3-hexenyl-acetate in oviposition site detection by *S. frugiperda* was further tested. Contrasting against rice exposed to hexane (Fig. 4A), the females showed a significantly greater oviposition rate for rice exposed to (*Z*)-3-hexenyl-acetate (58.92% vs. 41.07%, *P* < 0.001). Meanwhile, there was no significant difference in oviposition preference between maize and rice exposed to (*Z*)-3-hexenyl-acetate (maize: 49.83% vs. rice: 50.17%; *P* > 0.05) (Fig. 4B). These results suggested that (*Z*)-3-hexenyl-acetate mediates differential oviposition on maize and rice.

### Synchronization between oviposition rhythm of *S. frugiperda* and release rhythm of (*Z*)-3-hexenyl-acetate

**Fig. 5:**
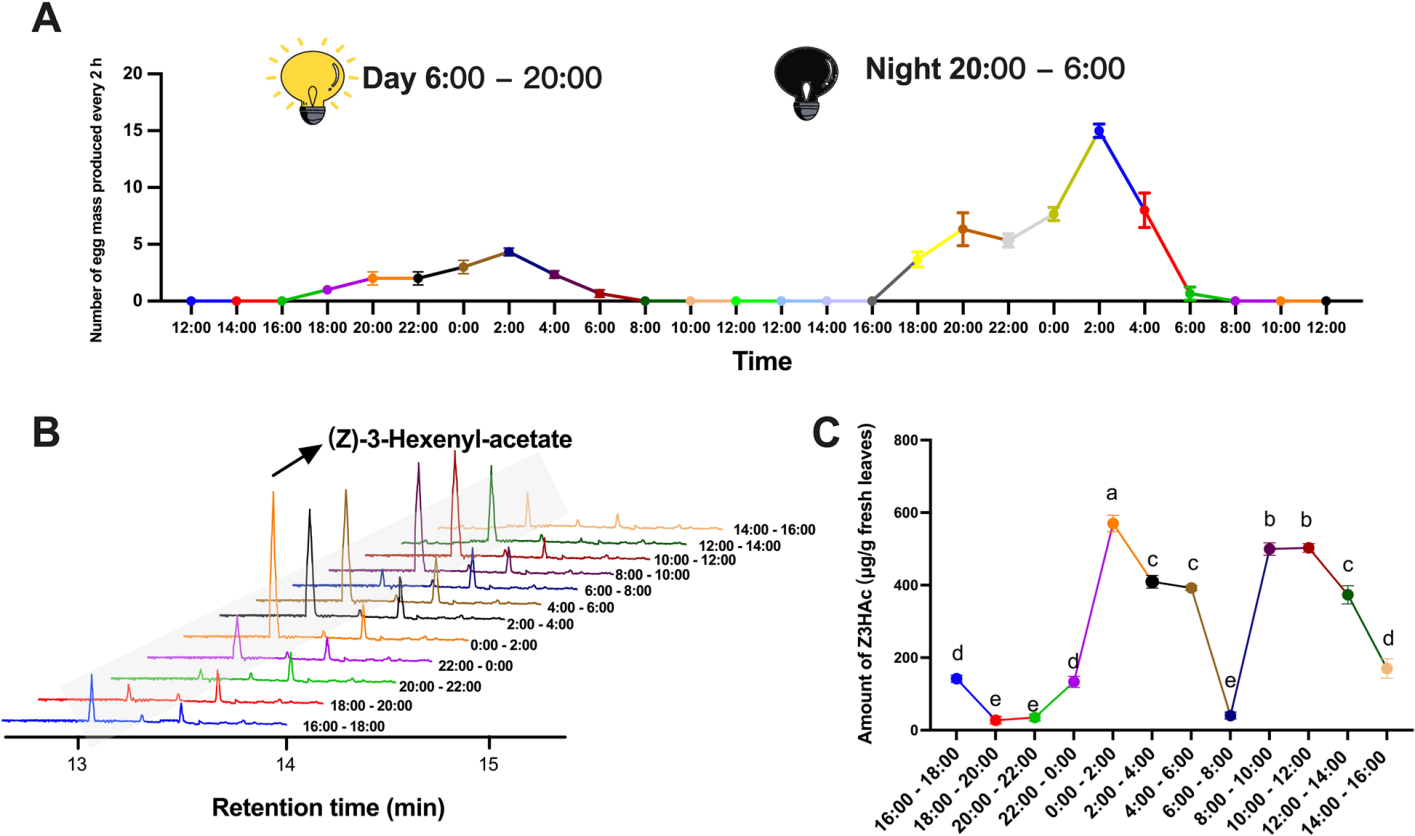
Time courses for oviposition by *S. frugiperda* females and release of (*Z*)-3-hexenyl-acetate by maize. **A** Oviposition by female *S. frugiperda* adults recorded every 2 h during a 48-h period. **B** Emissions of (*Z*)-3-hexenyl-acetate by maize seedlings over a 24-h collection period. **C** (*Z*)-3-hexenyl-acetate emission from maize over time. Comparisons between groups were made using ANOVA followed by Tukey’s test, *n* = 3. Error bars represent the SEM. Different letters above the error bars indicated a significant difference at the 0.05 level, the detailed data can be found in the “ Availability of Data and Materials”

To further verify the association between (*Z*)-3-hexenyl-acetate and the oviposition by female *S. frugiperda* adults, whether a correlation exists between the timing of oviposition peaks and the release pattern of (*Z*)-3-hexenyl-acetate was then investigated. The oviposition pattern by females was shown in Fig. 5A: 2-days-old *S. frugiperda* began laying eggs at 16:00 and stopped laying eggs after 06:00, with the number of egg masses peaked at 02:00. Additionally, the 3-days-old female *S. frugiperda* laid more eggs than the 2-day-old female *S. frugiperda*. Figure 5B and C displayed the emission pattern of (*Z*)-3-hexenyl-acetate every 2 h over a 24-h period. The production rate of (*Z*)-3-hexenyl-acetate peaked around 02:00, which was in coincidence with the peak time of oviposition.

### Field-trapping experiment

**Fig. 6:**
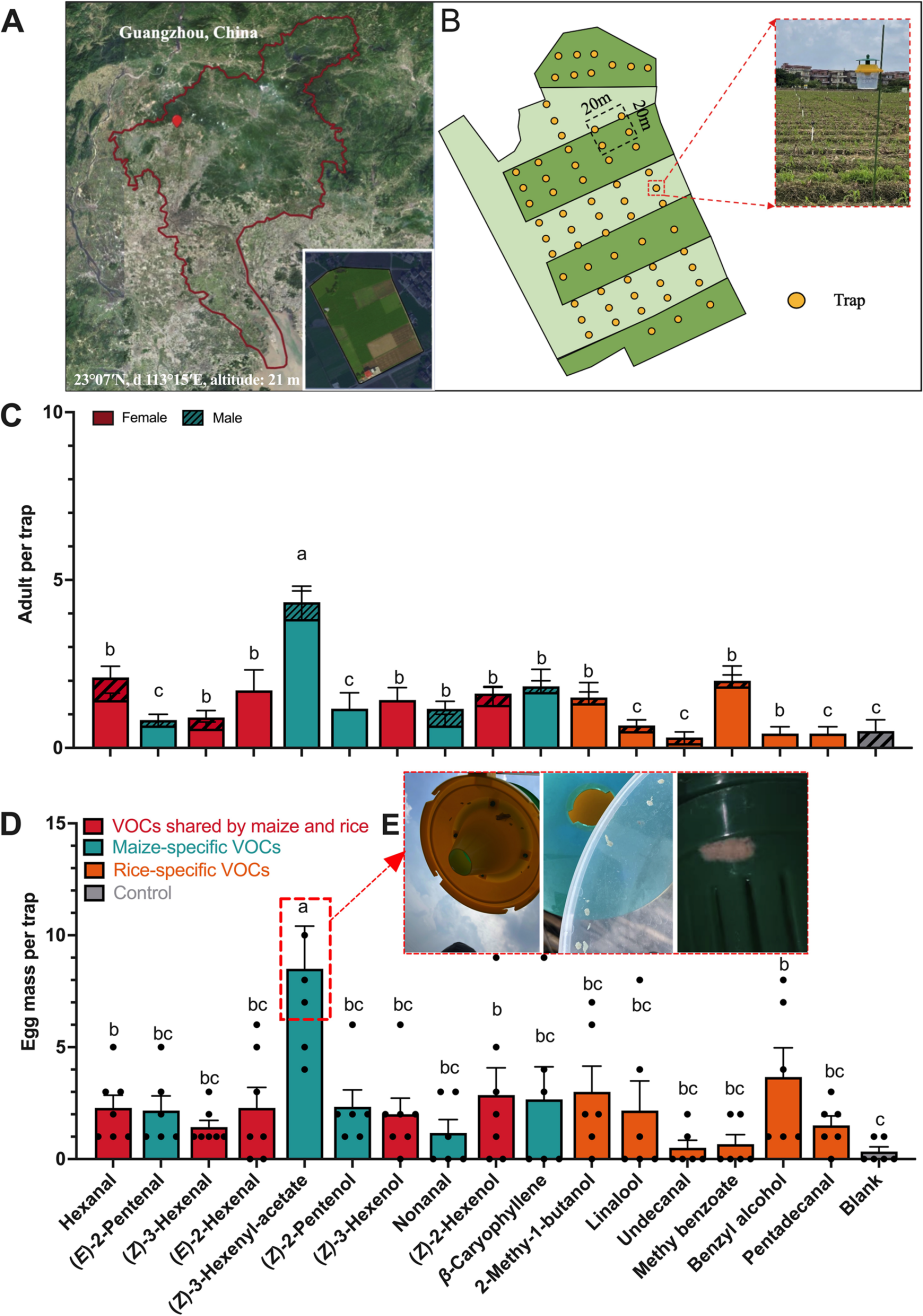
Oviposition by female *S. frugiperda* adults inside traps baited with individual VOCs in the field. **A** Map of the Guangzhou area with trap sites marked. Satellite image courtesy of Google Maps. **B** Schematic of field trapping experiment.** C** Numbers (mean ± SE) of female and male *S. frugiperda* captured by VOCs (Kruskal–Wallis test, *n* = 6; *P* < 0.05). Sixteen antennally-active compounds (100 μg) were tested individually. *n*-Hexane was used as blank control. **D** Number (mean ± SE) of egg masses deposited by *S. frugiperda* females inside traps (Kruskal–Wallis test, *n* = 6; *P* < 0.05). Sixteen antennally-active compounds (100 μg) were tested individually. *n*-Hexane was used as blank control. **E** The captured egg mass in a trap with (*Z*)-3-hexenyl-acetate

The effects of individual VOCs on the capture of *S. frugiperda* adults and the oviposition of female *S. frugiperda* adults were investigated in the field (Fig. 6A). Thirteen tested VOCs attracted significantly more *S. frugiperda* than the blank. Notably, (*Z*)-3-hexenyl-acetate baited trap captured the highest number of *S. frugiperda* adults (Fig. 6B). Furthermore, the number of egg masses in (*Z*)-3-hexenyl-acetate-baited traps was significantly higher than that in all other compound-baited traps (Fig. 6C). These results preliminarily verified that (*Z*)-3-hexenyl-acetate was able to attract *S. frugiperda* and stimulate females to oviposit in the field. These results preliminarily verified the role of (*Z*)-3-hexenyl-acetate in trapping *S. frugiperda* and stimulating oviposition in the field. Further extensive field-trapping experiments (multiple locations and years) are required to confirm the patterns.

### OR functional analysis with *Xenopus* oocyte expressing system

To gain insights into the molecular mechanisms of VOCs detection by *S. frugiperda*, 25 olfactory receptor genes that are known to be expressed in the antennae of *S. frugiperda* were successfully cloned (Fig. S3), which include an olfactory co-receptor (*SfruORco*) [57]. qRT-PCR analyses with the cloned ORs revealed that the expression of 2 ORs (namely *SfruOR23* and *SfruOR20*) was female-biased (Fig. S4).

**Fig. 7:**
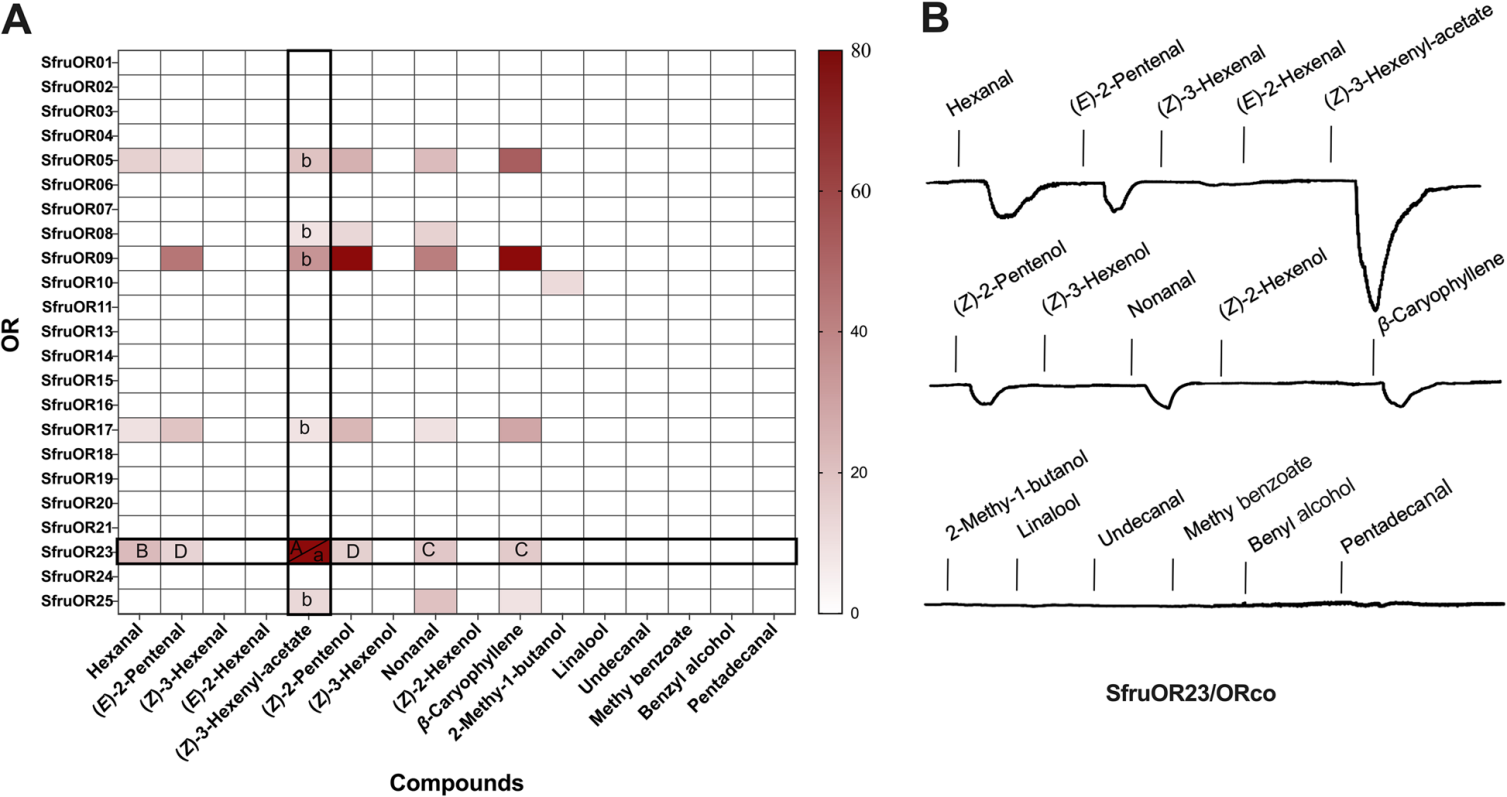
Receptor responses to VOCs. **A** Inward current responses of 24 olfactory receptors stimulated by the 16 VOCs (one-way ANOVA followed by Tukey’s pairwise test, *n* = 6, *P* < 0.05). **B** Representative two-electrode voltage-clamp traces of *SfruOR23/ORco* ectopically expressed in *Xenopus* oocytes

All twenty-five *S. frugiperda* ORs were then ectopically expressed and tested for responses to a panel of sixteen odorants using electrophysiological recording in the *Xenopus* oocyte system. In the OR-odorant matrix, sixteen out of twenty-five ORs did not respond to any of the VOCs (Fig. S5). For the other ORs in the antenna, most of them responded to maize-specific volatiles, while fewer ORs respond to rice-specific volatiles. The ORs *SfruOR5*, *SfruOR9*, *SfruOR17*, *SfruOR23*, and *SfruOR25* responded to the oviposition stimulant (*Z*)-3-hexenyl-acetate, with *SfruOR23*/ORco showing the highest response (Fig. 7A). Moreover, *SfruOR23* showed the highest responses to (*Z*)*-3*-hexenyl-acetate among all the volatile compounds (Fig. 7B). We therefore concluded that the key olfactory receptor of (*Z*)*-3*-hexenyl-acetate was *SfruOR23*.

### Effect of *SfruOR23* knockdown on the antennal response to VOCs

**Fig. 8:**
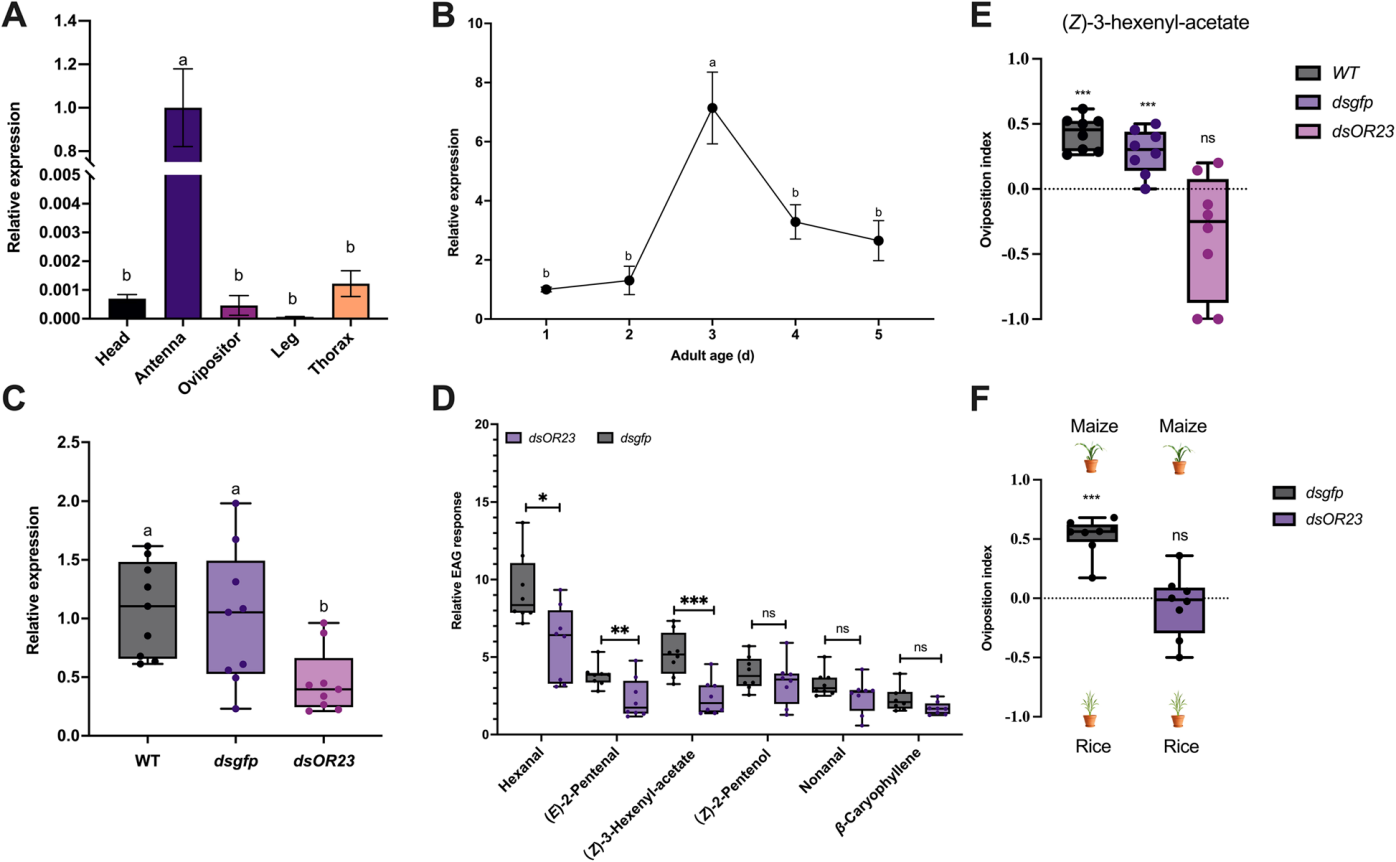
Expression patterns of *SfruOR23* and the effect of *SfruOR23* silencing on EAG response and oviposition preference*. ***A** Expression level of *SfruOR23* in different tissues of *S. frugiperda* (one-way ANOVA followed by Tukey’s pairwise test, n = 9, *P* < 0.05). **B** Time-course expression patterns of *SfruOR23* (one-way ANOVA followed by Tukey’s pairwise test, *n* = 9, *P* < 0.05). **C** mRNA expression level of *SfruOR23* 5 d after RNAi treatment (one-way ANOVA followed by Tukey’s pairwise test, *n* = 9, *P* < 0.05). **D** Effects of RNAi *SfruOR23* and *gfp* injection on EAG response to six VOCs (in one-sample *t*-test, *n* = 8; ns:* P* > 0.05, *: *P* < 0.05, **: *P* < 0.01, ***: *P* < 0.001). **E** Oviposition preference of wild type (WT), *dsgfp*, and *dsOR23* females in response to (*Z*)-3-hexenyl-acetate. Different letters above the bars indicate a significant difference (chi-square test, *n* = 8; ns: *P* > 0.05; ***: *P* < 0.001). **F** Oviposition preference of *dsgfp* and *dsOR23* females for maize and rice (Chi-Square Test,* n* = 8; ns: *P* > 0.05; ***: *P* < 0.001)

To further validate whether *SfruOR23* mediated the detection of oviposition stimulant (*Z*)*-3*-hexenyl-acetate, we knocked down *SfruOR23* in the female pupae of *S. frugiperda* by RNAi technique. First, the spatial and temporal expression of *SfruOR23* in *S. frugiperda* was investigated. The relative expression of *SfruOR23* was the highest in the antenna, in which the expression increased with the adult age (Fig. 8A and B). To investigate the RNAi efficiency, qRT-PCR assays were then performed. The injection of dsRNA decreased the expression level of the *SfruOR23* gene. Injection of *dsOR23* significantly decreased *SfruOR23* mRNA levels on the third day. The transcript level of *SfruOR23* was reduced to 56.11% for wild type and 49.54% for *dsgfp*, respectively (Fig. 8C).

The physiological function of *SfruOR23* in the perception of VOCs was evaluated by recording the EAG responses of RNAi-treated females. EAG activities of females in response to hexanal (*P* < 0.05), (*E*)-2-pentenal (*P* < 0.01), and (*Z*)-3-hexenyl-acetate (*P* < 0.001) were significantly reduced when *SfruOR23* was silenced by injection of *dsOR23*. There was no significant decrease in the responses to (*Z*)-2-pentenol, nonanal, and *β*-caryophyllene (Fig. 8D). Further two-choice oviposition assays same as 3.3.1 were conducted to investigate the behavioral responses of *dsOR23-*treated moths to (*Z*)-3-hexenyl-acetate and maize. The *dsgfp*-treated *S. frugiperda* females showed a preference for maize plants (*P* < 0.001). However, there was no significant oviposition preference for the *dsOR23*-treated females (*P* > 0.05) (Fig. 8E and F). The *SfruOR23* impaired groups appeared to have lost the most odorant-detecting capability. Therefore, we concluded that *SfruOR23* plays a crucial role in causing the oviposition preference of *S. frugiperda* for the host plants.

## Discussion

The females of *S. frugiperda* sense plant-derived VOCs to select a host for oviposition [41, 42]. The Chinese population of *S. frugiperda*, a hybrid of a corn strain and a rice strain, showed a distinct oviposition preference for maize plants over rice plants in the choice-oviposition experiment (Fig. 1B). To decipher the mechanism mediating the oviposition preference of *S. frugiperda*, we conducted a series of behavioral experiments. *S. frugiperda* showed a significant preference for rice or maize odors in Y-maze olfactory experiments. However, when given a choice between rice and maize plant odors, *S. frugiperda* showed no preference (Fig. 1D). These results suggest that the oviposition preference of *S. frugiperda* for maize plant over rice plant was not due to the attractiveness of the host plant odors. In the no-choice oviposition assay (Fig. 1F), significantly more eggs were laid on maize plants than on rice plants. These results confirm that host plant odors play a significant role in stimulating oviposition behavior. Previous studies have proven that host plant volatiles can stimulate oviposition behavior [47]. Further choice-oviposition assay with maize and rice homogenates demonstrated that *S. frugiperda* preferred maize homogenate for oviposition (Fig. 1H), indicating that the oviposition preference was mediated by VOCs. Taking the results together, we conclude that the simulation by VOCs from the maize plant accounts for the oviposition preference of *S. frugiperda*.

Female lepidopterans rely strongly on chemical stimuli, such as VOCs, to locate suitable host plants for oviposition [8, 58, 59]. A previous study showed that B73-*lox10* seedlings, which are deficient in VOC emissions, had fewer eggs laid by *S. frugiperda* [60]. In this study, we identified the VOCs that simulate the oviposition of *S. frugiperda.* Among the antennally active compounds identified by GC-EAD and GC–MS/MS, (*Z*)-3-hexenyl-acetate, a VOC specific to maize, was found to trigger oviposition behavior of female *S. frugiperda* on maize. Gravid females of *S. frugiperda* were found to show antennal response to (*Z*)-3-hexenyl-acetate in previous studies [43]. With additional behavioral assays, the correlation between (*Z*)-3-hexenyl-acetate and the oviposition behavior of female *S. frugiperda* was confirmed. The presence of (*Z*)-3-hexenyl-acetate in rice plants increased the oviposition preference of female *S. frugiperda* compared to untreated rice plants, and there was no difference in oviposition preference between maize plants and rice plants treated with (*Z*)-3-hexenyl-acetate (Fig. 4B). Thus, we concluded that the absence of (*Z*)-3-hexenyl-acetate in rice plants appears to be the reason for the oviposition preference of *S. frugiperda* for maize over rice plants. This study represents the first report identifying (*Z*)-3-hexenyl-acetate as a potential oviposition stimulant for *S. frugiperda.*

As *S. frugiperda* is known to oviposit during night-time [60], to explore whether the release rhythm of (*Z*)-3-hexenyl-acetate in maize is related to the oviposition rhythm of *S. frugiperda*, we carried out a synchronization experiment. The results of the experiment showed that the dark-phase release pattern of (*Z*)-3-hexenyl-acetate by maize plants was consistent with the oviposition dynamics of female *S. frugiperda* (Fig. 5). This finding has practical implications for controlling *S. frugiperda* populations, as traps baited with (*Z*)-3-hexenyl-acetate were found to be more effective in attracting *S. frugiperda* and receiving more egg masses than traps baited with other VOCs or a blank control (Fig. 6C). These findings provide further evidence of the role of (*Z*)-3-hexenyl-acetate in mediating the oviposition preference of *S. frugiperda.* In response to attacks by herbivores, maize plants produce a large amount of (*Z*)-3-hexenyl-acetate [61]. This compound alerts the nearby plants to activate their own defense against potential herbivore attack [62, 63], indicating the important role of (*Z*)-3-hexenyl-acetate in indirect defense. The addition of (*Z*)-3-hexenyl-acetate to traps containing sex pheromones can further increase their attractiveness to insects [64–70]. Thus, we identified (*Z*)-3-hexenyl-acetate as a unique volatile compound from maize that can be used as an oviposition attractant. It attracts mating females and improves the effectiveness of the *S. frugiperda* trap.

Olfactory receptors (ORs) selectively recognize and respond to odor molecules, ultimately triggering corresponding behaviors in insects [71]. Insect antennae are highly sensitive in detecting (*Z*)-3-hexenyl-acetate. In this study, twenty-four ORs and ORco identified from the antenna of *S. frugiperda* were ectopically expressed in the *Xenopus* oocyte system. Among them, only *SfruOR23* exhibited a strong response to (*Z*)-3-hexenyl-acetate, indicating it is a key olfactory receptor for this compound. The knockout of *SfruOR23* resulted in a significant reduction in antennal response to (*Z*)-3-hexenyl-acetate, together with the loss of oviposition preference to (*Z*)-3-hexenyl-acetate and maize in the ds*OR23* mutant line. These findings suggest that the sensitivity of *SfruOR23* to (*Z*)-3-hexenyl-acetate plays a crucial role in determining the oviposition preference of *S. frugiperda.* Moreover, *SfruOR23* has the potential to regulate oviposition. The 25 ORs were phylogenetically analyzed along with ORs from other Lepidopteran species with known ligands, including *Spodoptera litura* [72], *Spodoptera exigua* [73], *Helicoverpa armigera* [74], *Bombyx mori* Linnaeus [71], and *Helicoverpa assulta* [74]. It was observed that the ORs from different insects that detect (*Z*)-3-hexenyl-acetate formed three small clusters with each containing at least one odorant receptor, indicating conserved receptors for (*Z*)-3-hexenyl-acetate. *SfruOR23* and *SfruOR5* were clustered together with their ligands being consistent (Fig. S6). We speculated that *S. frugiperda* had undergone functional expansion in sensing (*Z*)-3-hexenyl-acetate. However, after RNAi knockdown of *SfruOR23*, weak antenna responses to (*Z*)-3-hexenyl-acetate were still observed, which indicate the possible compensation effect of *SfruOR8* for the function of *SfruOR23*. These findings together suggest that ORs can interact with each other to complete the encoding of odor. Screening of odor molecules that have strong binding affinities to ORs can contribute to the development of more effective oviposition attractants, leading to enhanced controlling effects and reduced financial losses caused by pests. In addition, it can enable the design of pesticides that target specific ORs to control pest populations.

## Conclusions

In summary, the oviposition preference of *S. frugiperda* was investigated using experiments including headspace collection, GC-EAD, GC–MS/MS, electrophysiological recordings, behavioral tests, and RNAi technology*.* The oviposition behavior of *S. frugiperda* was evaluated in maize and rice, and the mechanism was demonstrated to be the oviposition stimulation of a VOC other than the choice preference for host plants. (*Z*)-3-Hexenyl acetate is the key VOC responsible for oviposition stimulation by maize plants, and *SfruOR23* is the OR being sensitive to the specific oviposition stimulant. Our results indicate the molecular mechanism of (*Z*)-3-hexenyl acetate perception in *S. frugiperda* and provide molecular targets for the development of more efficient oviposition attractants.

## Methods

### Sources of insects and chemicals

*S. frugiperda* larvae were collected from a maize (Strain, Hua Meitian 8, from South China Agricultural University) field in Guangzhou, China (23°07′N, d 113°15′E, altitude: 21 m) and reared on an artificial diet [75] in the laboratory for over thirty generations, the average duration of each generation is about 30 days (*ca*. 26 °C, 70% relative humidity, LD 14:10 h). Our rearing population is large to fulfill the need of several ongoing projects with *S. frugiperda*, and wild individuals from the fields were introduced each year to enhance genetic diversity and mitigate the impact of inbreeding. The sex ratio was maintained at 1:1. The newly emerged adults were kept together in a nylon cage (40 cm × 40 cm × 40 cm) and provided with a 10% honey solution. Mated females were used for all laboratory tests. The commercial sources, CAS numbers, and purities of all standards/reagents are shown in Table S2 in the supplementary information.

### Two-choice oviposition preference test

To investigate the oviposition preference of female *S. frugiperda* adults to maize and rice plants, we performed a two-choice oviposition assay in a dark room. The maize (Strain, Hua Meitian 8) and rice (Strain, Zhonghua 11) plants were grown in a greenhouse at 26 °C, with 60% relative humidity and LD 16:8 h. A potted maize seedling at the four-leaf stage and a potted rice seedling at the tillering stage about 30 cm high were cultivated under laboratory conditions (*ca.* 26 °C, 70% relative humidity, LD 14:10 h). Seedlings were allowed to acclimate to the laboratory conditions for 3 days, the “maize model” and “rice model” are plant models made of silk cloth and are very similar to real plants (at the same height and color as maize or rice seedlings). They were used as a control to eliminate the visual difference from *S. frugiperda*. Thirty pairs of newly emerged (1-day-old) female and male moths were released into the cage (Fig. 1A). After 3 days, the amounts of egg masses on all plant leaves were counted. The experiment was replicated ten times. The oviposition index was calculated as (T − C)/(T + C) where T is the number of egg masses on the maize or rice seeding side and C is the number of egg masses on the maize or rice model seeding.

### Two-choice olfactory assays of host plant

To investigate whether the oviposition preference of *S. frugiperda* is caused by the choice preference of olfactory or oviposition stimulation, first, we conducted a Y-tube olfactometer (stem, 25 cm; arms, 20 cm at the angle of 60°; internal diameter, 10 cm) assay. The maize and rice plant are the same as above. A potted maize and rice plant (30 cm high) was enclosed in a 2.5 L polyethylene terephthalate bag (Fig. 1B). The pushing airflow rate was 500 mL/min and controlled by a vacuum pump connected to the two arms of the olfactometer through activated charcoal filters. To start a measurement, a mated female moth was placed at the base of the main arm, and its behavior was observed for 5 min under a 0.61 Lux red lamp in a dark room [5]. Each female moth had a choice between the treatment and the control. A female was considered to have made the choice when it moved > 5 cm into either arm (visually assessed by a line marked on both arms) and stayed for at least 30 s. Each treatment was replicated 30 times. The position of treatment and control was reversed after every five individuals and was replaced with a cleaned Y-tube after 10 tests. Y-tubes were cleaned with acetone and oven-dried at 100 °C for at least 2 h prior to use.

### No-choice oviposition stimulate test

We tested the oviposition-stimulating activity of oviposition-preferring maize and rice plant in a small cage (40 cm × 40 cm × 40 cm). The oviposition-preferred maize and rice or model plants (control) described above were then placed in the middle of the cage. A pair of newly emerged female and male moths were put into the cage exposed to the maize and rice plant for 96 h (Fig. 1E). A 10% honey solution was provided for adult feeding. Egg masses being deposited inside each treatment and the control was counted every 12 h starting from 24 to 96 h after treatment. Each treatment was replicated 30 times.

### Two-choice oviposition preference of homogenate

To investigate whether the oviposition preference of *S. frugiperda* is due to VOCs, we measured the oviposition preference of females for the maize and rice leaf homogenate by using a two-choice assay in a cage. We homogenized maize and rice 100 g leaves with 100 mL water, the model plant was dipped the homogenate into individually, and the control was treated with the same volume of water on the model plant. Other conditions were conducted with the same methods as mentioned above for the oviposition preference test.

### Host plant volatile collection and chemical analyses

Fresh maize and rice leaf blades without pest damage (about 0.1 kg) were placed in a glass vessel (15 cm in diameter, 20 cm in height) for headspace extraction. An airflow (500 mL/min) generated by a vacuum pump (QC-1S; Beijing Municipal Institute of Labor Protection, Beijing, China) was purified with activated charcoal and pumped into the vessel. Then the airflow passed through an absorbing column for 3 h. A control was conducted by passing an air through empty glass vessel to exclude possible contamination. Prior to volatiles collection, absorbing columns (a glass filter, ID: 4 mm; containing 200 mg Porapak-Q, 80/100 mesh, Supelco, Bellefonte, PA, USA) used in the experiments were cleaned sequentially with 3 mL methanol and dichloromethane (DCM), and then exposed in a flow of dry nitrogen at 180 °C for 30 min. Adsorbed volatiles by the Porapak-Q column was eluted with 1 mL of DCM. The extract was concentrated to 200 μL with a nitrogen-blowing instrument and kept at − 20 °C for further use in GC-EAD and GC/MS/MS analyses.

### GC-EAD

The extraction was analyzed by a Shimadzu GC-2010plus equipped with a flame ionization detector (FID) and a polar DB-Wax column (30 cm × 0.25 mm × 0.25 μm, Agilent, USA). The experimental details were described by Zhang et al. [76]. In short, nitrogen (2.0 mL/min) was used as carrier gas. The oven temperature was kept at an initial of 40 °C for 4 min, programmed at 3 °C/min to 80 °C, then 15 °C/min to 240 °C, and held for 2 min. The column effluent was split at the end with a 1:2 ratio, with one-third to a heated line into a humidified airstream (400 mL/min) which was directed to the antenna, and the other two-thirds directed to FID. The FID signal was recorded on a computer using GC-EAD Pro software (version 4.4, Syntech, Kirchzarten, Germany).

An antenna from a 2-day-old female was carefully cut off with the tip excised. The base of the antenna was connected to the reference glass electrode (outer diameter 1.5 mm, inter diameter 0.84 mm, Vital Sense Scientific Instruments Co. Ltd., Chengdu, China) while the antennal tip was brought into contact with the recording glass electrode. Both electrodes were prefilled with a Beadle Ringer's solution modified by Tween^®^80 (0.05%, W/V), and platinum wires were used to maintain the current between the antennal preparation and a Syntech EAG Combi probe [77].

### GC–MS/MS

Maize and rice volatile samples were analyzed on a Shimadzu TQ8050 NX GC–MS/MS fitted with a polar DB-Wax capillary column with the same dimension as described above. Helium (1.0 mL/min) was the carrier gas, 1 µL of the samples was injected in spitless mode at 230 °C. The oven temperature was programmed as GC-EAD analysis. The temperature of the transfer line was set at 250 °C. The EAD active compounds were tentatively identified by comparison with the NIST08 MS library, and the retention times and mass spectra of identified compounds were confirmed through injecting synthetics under the same GC–MS/MS program as described above, verified by injecting a mixture containing 100 ng of authentic standard.

### Two-choice oviposition assay with antennally-active compounds

To investigate which female antennally-active compounds induce oviposition preference in *S. frugiperda*, a two-choice assay was conducted in a small cage (40 cm × 40 cm × 40 cm) (Fig. 3A). Fifteen pairs of 1-day-old female and male moths were released into the cage. A small Petri dish with a 10% honey solution was placed in the middle of the bottom of the cage for adult feeding. The antennally active compounds (10 μg, diluted with *n*-hexane) or solvent control (*n*-hexane) were loaded onto a bell-shaped septum (length = 1.5 cm, Beijing Pherobio Technology Co., Ltd., Beijing, China). Two days post introduction of moth pairs, the septum with individual test compounds or solvent was fixed on the opposite inner sides of the cage. Egg masses deposited on the treatment and control sides were counted 24 h after volatile exposure. Each experiment was replicated eight times. The oviposition index was calculated as (T − C)/(T + C) where T is the number of egg masses on the treatment side and C is the number of egg masses on the control side.

### Two-choice olfactory assays with antennally-active compounds

To investigate whether the oviposition preference of *S. frugiperda* is due to attraction by the oviposition-preferring compound, we conducted a Y-tube olfactometer and the experimental environmental conditions described above. The oviposition-preferring compounds were diluted with hexane to achieve three dose, 1, 10, and 100 μg. Ten microliters of each solution were loaded onto filter paper strips. The filter paper strip with the test compound was then placed into one arm of the olfactometer while the hexane-treated filter paper strip was placed into the other arm. Each treatment was replicated 30 times.

### No-choice olfaction oviposition stimulating assays

To test the oviposition-stimulating activity of oviposition-preferring VOCs screened in *2.4.1*, oviposition-stimulating assays were conducted in 500-mL transparent plastic containers. The oviposition-preferring compound (10 μg, diluted with *n*-hexane) or *n*-hexane (control) was loaded onto a septum as described above, which was then placed in the plastic container. A pair of newly emerged female and male moths were into the container to expose to the oviposition-preferring active compound for 96 h. A 10% honey solution was provided for adult feeding. Egg masses being deposited inside each treatment and the control containers were counted every 12 h starting from 24 to 96 h after treatment. Each treatment was replicated 90 times.

### Effect of (*Z*)-3-hexenyl-acetate on oviposition preference

The oviposition preference of the females for (*Z*)-3-hexenyl-acetate-exposed rice was tested using the following method. (*Z*)-3-Hexenyl-acetate (10 μg, diluted with *n*-hexane) was loaded onto a septum. The control septum was treated with the same volume of *n*-hexane. In experiment I, a rice plant at tillering stage with (*Z*)-3-hexenyl-acetate treated septum and a maize plant at the four-leaf stage were placed in the big cage. In experiment II, a rice plant at tillering stage with (*Z*)-3-hexenyl-acetate treated septum and the other rice plants at the same stage were placed in the big cage described above. Both experiments were conducted at the same temperature and relative humidity as mentioned above for the oviposition preference test. Thirty pairs of newly emerged (1-day-old) female and male moths were released into the cage. The number of egg masses on each plant seedling was counted 3 days after moth introduction. Each experiment was replicated eight times.

### Synchronization between oviposition rhythms of *S. frugiperda* and release rhythm of (*Z*)-3-hexenyl-acetate

To further explore synchronization between them, we investigated the oviposition rhythm of *S. frugiperda* and the release rhythm of (*Z*)-3-hexenyl-acetate by maize plants. Fifteen pairs of 1-day-old female and male moths were released into the small cage as described above. The number of egg masses was counted every 2 h for a continuous 48 h starting from 12 p.m. After completing the second day of counting, the egg masses were gently brushed away with a fine brush without damaging the leaves. The experiment was replicated 3 times.

A potted maize plant (30-cm high) was enclosed in a 2.5-L polyethylene terephthalate bag. Charcoal-filtered air was pumped into the bag at 550 mL/min and the leaf odors were drawn out at 500 mL/min through a Porapak-Q (200 mg) filter. Headspace volatile collections were conducted continuously 24 h. Every 2 h the filter was replaced with a new one. The leaf volatiles captured by the Porapak-Q filters were eluted with DCM and analyzed on a capillary GC column (same as described above). Authentic (*Z*)-3-hexenyl acetate was used as an external standard to calibrate the GC peak area and quantify the amount present in each headspace sample.

### Field experiments

Then we evaluated the effectiveness of individual antennally-active volatiles on oviposition of female *S. frugiperda*, the numbers of adult moths captured, and the egg masses on VOCs and control trap (n-hexane) (Fig. 5A). A field experiment was conducted in a maize (new leaf stage to tassel stage) field in Guangzhou, China (23°07′N, 113°15′E, altitude: 21 m) from August 21 to September 20, 2021. Individual EAD-active compounds (100 mg) were loaded into a bell-shaped septum that was attached to a bucket-type trap (Beijing Pherobio Technology Co., Ltd., Yangling, China). Traps were hung on iron stakes at 20 cm above the maize seedlings and spaced 20 m apart in a randomized complete block design, with 6 replicates. The adults captured by, and the numbers of egg masses deposited on each trap were recorded and the traps were emptied every 3 days. The lures were replaced with new ones every 6 days. The blank septum served as the control.

### RNA extraction and cDNA preparation

To screen odorant receptors involved in the detection of the sixteen VOCs, female and male antennae were collected respectively for gene cloning. The collected samples were frozen in liquid nitrogen and stored at − 80 °C until use for RNA extraction. To determine the transcript level of candidate OR genes, total RNA was extracted by the Trizol method (TaKaRa, Japan) following the manufacturer’s protocol. RNA quality was determined by spectroscopy (Biowave II) and electrophoresis. The cDNAs were synthesized using the PrimeScriptTMRT reagent Kit and gDNA Eraser kit (TaKaRa, Japan).

### Full-length gene cloning

Previously, twenty-seven candidate ORs have been found from the antennal transcriptome of *S. frugiperda* [78]. To verify these reported ORs, we first aligned and compared all ORs’ amino acid sequences using ClustalW to remove the repetitive sequences and obtained twenty-seven partial-length ORs. Then we carried out RACE PCR was performed using a Smarter 5′/3′ Kit (TaKaRa, Japan) for 5′ ends amplification obtained twenty-six full-length sequences. The full-length sequences were assembled based on antennal transcriptome and RACE results and then confirmed by end-to-end PCR using specific primers designed at both ends. All the primers were designed by Primer Premier 5.0 (PREMIER Biosoft International, CA, USA) and listed in Table S3. The reactions were performed in 25 μL with 1 μL of cDNA, *PrimeStar* 12.5 μL, and 0.4 mM for each primer (TaKaRa, Japan). The PCR reactions for the full-length sequences were carried out under the following conditions: 98 °C for 2 min, 32 cycles of 98 °C for 30 s, 56 °C for 30 s, 72 °C for 1.5 min, and 72 °C for 5 min. PCR products were analyzed by electrophoresis on a 1% agarose gel. Target bands were purified and choned into pEASY-Blunt cloing vector (TransGen Biotech, Beijing, China) and sequenced at BGI Company (Shenzheng, China). The full-length open-reading frames of these genes were predicted using the open-reading frames finder. We successfully verified 25 OR genes, including the *SfruORco* [57] and 24 ORs.

### Vector construction and cRNA synthesis

The candidate OR genes and the *SfruORco* gene were amplified using the specific primers (Table S3). pT7TS expression vector with a cutting site of *SphI* or *SpeI*, using the ClonExpress® One Step Cloning Kit (TaKaRa, Japan). The confirmed plasmid and pT7TS expression vector were ligated with ClonExpress One Step Cloning Kit (Vazyme Biotech Co., Ltd). The plasmids were extracted by Plasmid Mini KitI (OMEGA biotek, USA). Purification of plasmid by restriction enzyme (*Smal/BamHI*) and as the templates to synthesize cRNAs by mMESSAGE mMACHINE® T7 Kit (Thermo Fisher Scientific, Waltham, MA, USA). Purifications of cRNAs were diluted with nuclease-free water at a concentration of 1 μg/μL and stored at − 80 °C until use.

### Receptor expression in *Xenopus oocytes* and two-electrode voltage clamp electrophysiological recordings

The full-length open-reading frames of OR genes were expressed in *X. laevis* oocytes, and the oocytes were analyzed using two-electrode voltage clamping, as previously reported [23]. The 1 M stock solutions of volatile compounds from maize and rice in dimethyl sulfoxide were prepared and stored at − 20 °C. The stock solution was diluted in Ca^2+^-free standard oocyte 10 × Ringer buffer (56.1 g NaCl, 1.5 g KCl, 2.1 g MgCl_2_·6H_2_O, 11.9 g HEPES, pH 7.6) prior to use. Six (replicates) were tested in the screening tests and the dose–response tests. Oocytes injected with sterilized ultrapure H_2_O were used as controls. We normalized the responses of each OR in each system by calculating the rate of the response to a compound to the average response to the most active compound.

### dsRNA synthesis and injection

To confirm the function of *SfruOR23* in the oviposition of females, the expression of *SfruOR23* was knocked down by in vitro injection of dsRNA to female pupa. Specific dsRNA primers of *SfruOR23* and *GFP (*Green fluorescent protein) (GenBank accession no: MN623123.1) containing a T7 promoter on the 5′ end were designed (Table S3). The 413-bp *SfruOR23* transmembrane domain and 289-bp *GFP* fragments were cloned with T7 promotor primers to facilitate the further synthesis of dsRNA. PCR products were loaded onto 1% agarose gel to check the target band using Gel Extraction Kit D2500 (OMEGA biotek, USA). The purified DNA was used as the template to synthesize the corresponding dsRNA using in vitro Transcription T7 Kit (TaKaRa, JAPAN) by incubating at 37 °C for 4 h. Finally, dsRNA quality was checked by electrophoresis and using a spectrometer (Biowave II).

First, to determine the expression level of *SfruOR23* in different adult tissues and 5 days expression pattern after eclosion, the head antennae, ovipositor, legs, and thorax, and samples were collected from female adults after 1 to 5 days, respectively. The total RNA of collected samples was extracted for qRT-PCR to detect the expression of *SfruOR23*. dsRNA (2 μg) or *GFP* was immediately injected into an *S. frugiperda* pupa on the tenth day of the pupal stage using a 10-μL microinjector (Gaoge Industry and Trade Co., Ltd., Shanghai). The injection position was between the third and fourth abdominal sternites. RNAi efficacy was assessed by qPCR after the *S. frugiperda* had eclosion for 3 d. The expression profiles of the gene were determined using the 2^−ΔΔCt^ method [79], the *S. frugiperda β*-Actin was utilized as the housekeeping reference gene [80].

### Electroantennography (EAG) responses and behavior verification after RNAi

For the EAG assays to record antennal responses to volatile compounds, female antennae from dsRNA were cut off on 3-day-old. The protocol for EAG assays is as previously described [81, 82]. *n*-Hexane was used as control. Filter paper strip (3 cm × 0.6 cm) impregnated with 10-μg test compound was allowed to evaporate solvent for 15 s, and then inserted into a Pasteur pipette to constitute an odor cartridge. The test stimuli were presented in a randomized order with 2-min intervals between two control puffs. The EAG responses to the test stimuli were corrected by subtracting the average amplitude of the two control signals. Each experiment was repeated 10 times.

The oviposition preference for (Z)-3-hexeneyl-acetate was tested in females in which the olfactory gene was silenced. we used the female pupae that were injected with either *dsOR23* (2 μg) or *dsgfp* (2 μg) on the DAY-9 before eclosion. After eclosion, fifteen injected females were put in small cages, and fifteen wild-type males were added in each cage as follows (in biological duplicates): (I) *dsgfp* female × wild type male, (II) *dsOR23* female × wild type male. Mixed sex groups were further visually observed to confirm the successful mating and placed in cages before the start of photophase. Two days after dsRNA application, the effect of (*Z*)*-*3*-*hexenyl-acetate was tested and the number of egg masses during a 24-h period was counted. Each treatment was replicated eight times.

### Statistical analysis

All data were analyzed by Prism 9 and SPSS 20.0. The data from oviposition and Y-tube olfactometer assays were analyzed by Chi-square (ns *P* > 0.05, *P < 0.05, ***P < 0.001). For the laboratory dual-choice bioassay and EAG amplitude measurements, data were analyzed by one-way ANOVA followed by Tukey’s HSD test, *P* < 0.05. Trap catch data among the treatments did not conform to Poisson distribution and were analyzed by the Kruskal–Wallis test (*P* < 0.05).

## Supplementary Information

**Additional file 1: Table S1.** The number of haplotypes in samples identified using *CO I* and *Tpi* marker.

**Additional file 2: Figure S1.** Olfactory preferences of female *S. frugiperda* to homogenates. Behavioral responses of female *S. frugiperda* to the maize homogenate *vs*. control, rice homogenate *vs*. control, and maize homogenate *vs*. rice homogenate (control: water, Chi-Square test, *n* = 30; ns, *P* > 0.05; *P < 0.05; ** *P* < 0.01; *** *P* < 0.001).

**Additional file 3: Table S2.** Chemicals used in experiments.

**Additional file 4. Figure S2.** Oviposition preference of female *S. frugiperda* to the antennally-active volatile compounds at different doses. A. Binary-choice tests for corn-specific VOCs vs. control. B. Binary-choice tests for rice-specific-VOCs vs control. C. Binary-choice tests for VOCs shared by rice and corn vs. control (Chi-square test, *n* = 8; ns, *P* > 0.05; *: *P* < 0.05; **: *P* < 0.01; ***: *P* < 0.001; gray: no difference at any concentration; red: significant attraction at one of the concentrations; green: significant aversion at one of the concentrations).

**Additional file 5: Figure S3.** Gene amplification of SfruORs and verification of recombinant plasmids PT7GS/ORs using PCR. M, 2000 marker. The numbers 1 to 25 represent the cloning of the *SfruOR1-SfruOR25* genes (except for *SfruOR12*). The numbers 26 to 50 represent the recombinant plasmids *PT7OR1-PT7OR25* (except for *PT7OR12*).

**Additional file 6: Figure S4.** OR gene expression in male and female antennae by quantitative real-time PCR (qRT-PCR). In one-sample *t*-test, *n* = 9; *: *P* < 0.05, **: *P* < 0.01, ***: *P* < 0.001 (green: Male; red: Female). qRT-PCR was used for expression analysis of the *SfruORco* gene and candidate OR genes to compare the transcription levels of each OR gene between the two sexes. The primers reported by Qiu *et al*. [78] were used (Table S3).

**Additional file 7: Figure S5.** Two-electrode Voltage-clamp Recordings in *Xenopus* oocytes. A. None of the antenna ORs responded to the 16 VOCs (10^-4^ M). *n* = 6 (oocytes). B. Inward current responses of *Xenopus* oocytes expressing ORs to VOCs (10^-4^ M). *n* = 6 (oocytes).

**Additional file 8: Figure S6.** Phylogenetic relationship of the odorant receptors in five *Spodoptera* species. The sequences of five lepidopterans, *Spodoptera litura* [72], *Spodoptera exigua* [73], *Helicoverpa armigera* [74], *Bombyx mori* [71], and *Helicoverpa assulta* [74]. ORs were retrieved from the NCBI website (http://www.ncbi.nlm.nih.gov). The 25 candidate OR genes in *S. frugiperda* are all clustered in the OR clade, using the sequences from antennal transcriptome data [78]. ClustalW alignment of the amino acid sequences was conducted with Bioedit v7.2 (https://bioedit.software.informer.com/7.2/). The maximum likelihood phylogenetic tree was constructed with 1000 bootstrap replicates using MEGA 6 (https://www.megasoftware.net/) based on amino acid sequence alignment and was further refined with iTOL v5 (https://itol.embl.de/). The OR receptor branches sensitive to-3-hexenyl-acetate of *S. frugiperda* (red and bold) and the branch sensitive to (*Z*)-3-hexenyl-acetate of other species (blue and bold) are indicated.

**Additional file 9: Table S3.** Primers used for RACE-clone, full-length clone, cRNA synthesis, RNA interference, and qRT-PCR.

## Data Availability

All data generated or analyzed during this study are included in this published article, and the datasets supporting the conclusions of this article are available in 10.6084/m9.figshare.23264939.

## Abbreviations

FAW: Fall armyworm
VOCs: Volatile organic compounds
*S.** frugiperda*: *Spotoptera frugiperda*
GC-EAD: Gas chromatography-electroantennographicdetection
GC–MS/MS: Gas chromatography-triple quadrupole mass spectrometry
RNAi: RNA interference
qRT-PCR: Real-time quantitative reverse transcription PCR
DCM: Dichloromethane

## References

[1] Knudsen GK, Bengtsson M, Kobro S, Jaastad G, Hofsvang T, Witzgall P (2008). Discrepancy in laboratory and field attraction of apple fruit moth *Argyresthia conjugella* to host plant volatiles. Physiol Entomol.

[2] Bruce TJ, Wadhams LJ, Woodcock CM (2005). Insect host location: a volatile situation. Trends Plant Science.

[3] Singer M, Vasco D, Parmesan C, Thomas C, Ng D (1992). Distinguishing between ‘preference’and ‘motivation’in food choice: an example from insect oviposition. Anim Behav.

[4] Baur R, Feeny P, Städler E (1993). Oviposition stimulants for the black swallowtail butterfly: identification of electrophysiologically active compounds in carrot volatiles. J Chem Ecol.

[5] Molnár BP, Tóth Z, Fejes-Tóth A, Dekker T, Kárpáti Z (2015). Electrophysiologically-active maize volatiles attract gravid female European corn borer, *Ostrinia nubilalis*. J Chem Ecol.

[6] Lin CC, Prokop Prigge KA, Preti G, Potter CJ (2015). Food odors trigger *Drosophila* males to deposit a pheromone that guides aggregation and female oviposition decisions. Elife.

[7] Knight A, Light D (2004). Use of ethyl (*E*, Z)-2,4-decadienoate in codling moth management: stimulation of oviposition. J Entomol Soc Br Columbia.

[8] Pagadala Damodaram KJ, Kempraj V, Aurade RM, Venkataramanappa RK, Nandagopal B, Verghese A (2014). Oviposition site-selection by *Bactrocera dorsalis* is mediated through an innate recognition template tuned to γ-octalactone. PLoS one.

[9] Kamala Jayanthi PD, Kempraj V, Aurade RM, Venkataramanappa RK, Nandagopal B, Verghese A (2014). Specific volatile compounds from mango elicit oviposition in gravid *Bactrocera dorsalis* females. J Chem Ecol.

[10] Joseph RM, Devineni AV, King IF, Heberlein U (2009). Oviposition preference for and positional avoidance of acetic acid provide a model for competing behavioral drives in *Drosophila*. Proc Natl Acad Sci.

[11] Janz N. Evolutionary ecology of oviposition strategies. Chemoecol Insect Eggs Egg Deposition. 2003:349–76.

[12] Courtney SP, Chen GK, Gardner A. A general model for individual host selection. Oikos. 1989:55–65.

[13] Thompson JN (1988). Evolutionary genetics of oviposition preference in swallowtail butterflies. Evolution.

[14] Renwick J (1989). Chemical ecology of oviposition in phytophagous insects. Experientia.

[15] Thompson JN, Pellmyr O (1991). Evolution of oviposition behavior and host preference in Lepidoptera. Annu Rev Entomol.

[16] Jaenike J (1990). Host specialization in phytophagous insects. Annu Rev Ecol Syst.

[17] Knolhoff LM, Heckel DG (2014). Behavioral assays for studies of host plant choice and adaptation in herbivorous insects. Annu Rev Entomol.

[18] Thompson JN (1988). Evolutionary ecology of the relationship between oviposition preference and performance of offspring in phytophagous insects. Entomol Exp Appl.

[19] Tabashnik BE, Wheelock H, Rainbolt JD, Watt WB (1981). Individual variation in oviposition preference in the butterfly, Colias eurytheme. Oecologia.

[20] Blanco CA, Pellegaud JG, Nava Camberos U, Lugo Barrera D, Vega Aquino P, Coello J (2014). Maize pests in Mexico and challenges for the adoption of integrated pest management programs. J Integr Pest Manag.

[21] Goergen G, Kumar PL, Sankung SB, Togola A, Tamò M (2016). First report of outbreaks of the fall armyworm *Spodoptera frugiperda* (JE Smith)(Lepidoptera, Noctuidae), a new alien invasive pest in West and Central Africa. PLoS one.

[22] Stokstad E. New crop pest takes Africa at lightning speed. In: American Association for the Advancement of Science. 2017:473–474. 10.1126/science.356.6337.473 28473543

[23] Montezano DG, Sosa Gómez D, Specht A, Roque Specht VF, Sousa Silva JC, Paula Moraes SD (2018). Host plants of *Spodoptera frugiperda* (Lepidoptera: Noctuidae) in the Americas. Afr Entomol.

[24] Devi S (2018). Fall armyworm threatens food security in southern Africa. Lancet.

[25] Lestari P, Budiarti A, Fitriana Y, Susilo F, Swibawa IG, Sudarsono H, et al. Identification and genetic diversity of *Spodoptera frugiperda* in Lampung Province, Indonesia. Biodiver J Biol Diver. 2020;21(4).1670–7.

[26] Supartha IW, Susilau IW, Sunari AAAAS, Mahaputra IF, Yudha IKW, Wiradana PA. Damage characteristics and distribution patterns of invasive pest, *Spodoptera frugiperda* (JE Smith)(Lepidoptera: Noctuidae) on maize crop in Bali, Indonesia. Biodiver J Biol Divers. 2021;22(6).

[27] Chen H, Yang XL, Chen AD, Li YC, Wang DH, Liu J (2020). Immigration timing and origin of the first fall armyworms (*Spodoptera frugiperda*) detected in China. Chin J Appl Entomol.

[28] Wu QL, He LM, Shen XJ, Jiang YY, Wu KM (2019). Estimation of the potential infestation area of newly-invaded fall armyworm *Spodoptera frugiperda* in the Yangtze River Valley of China. Insects.

[29] Yang XL, Liu YC, Luo MZ, Li Y, Wang WH, Wan F (2019). Fall armyworm was firstly detected in Jiangcheng County, Yunnan, China. Yunnan Agriculture.

[30] Center NATES (2019). Major pest Spodoptera frugiperda have invaded in Yunnan, and all areas should immediately strengthen investigation and monitoring.

[31] Sun XX, Hu CX, Jia HR, Wu QL, Shen XJ, Zhao SY (2021). Case study on the first immigration of fall armyworm, *Spodoptera frugiperda* invading into China. J Integr Agric.

[32] Jiang YY, Liu J, Xie MC, Li YH, Yang J, Zhang ML (2019). Observation on law of diffusion damage of *Spodoptera frugiperda* in China in 2019. Plant Prot.

[33] Meagher RL, Nagoshi RN (2012). Differential feeding of fall armyworm (Lepidoptera: Noctuidae) host strains on meridic and natural diets. Ann Entomol Soc Am.

[34] Pashley DP (1986). Host-associated genetic differentiation in fall armyworm (Lepidoptera: Noctuidae): a sibling species complex?. Ann Entomol Soc Am.

[35] Nagoshi RN, Meagher RL (2004). Behavior and distribution of the two fall armyworm host strains in Florida. Florida Entomol.

[36] Cock MJ, Beseh PK, Buddie AG, Cafá G, Crozier J (2017). Molecular methods to detect *Spodoptera frugiperda* in Ghana, and implications for monitoring the spread of invasive species in developing countries. Sci Rep.

[37] Zhang L, Jing MH, Zhang DD, Jiang YY, Liu J, Wu KM (2019). Molecular identification of invasive fal armyworm *Spodoptera frugiperda* in Yunnan Province. Plant Prot.

[38] Zhang ZT, Xie AT, Dong J, Yang JG, Zhang AH, Wang SY (2021). Identification of *Spodoptera frugiperda* host strains in Beijing and other regions. China Plant Protect.

[39] Wang JL, Wei JQ, Sun ZX, Xu HH, Lin F (2020). Haplotype and genetic diversity analysis of *Spodoptera frugiperda* invading in three provinces of central and southern China. J South China Agric Univ.

[40] Qiu ML, Liu QQ, Yang XJ, Huang XY, Guan RF, Liu BP (2020). Feeding and oviposition preference and fitness of the fall armyworm, *Spodoptera frugiperda* (Lepidoptera: Noctuidae), on rice and maize. Acta Entomol Sin.

[41] Yactayo Chang JP, Mendoza J, Willms SD, Rering CC, Beck JJ, Block AK (2021). *Zea mays* volatiles that influence oviposition and feeding behaviors of *Spodoptera frugiperda*. J Chem Ecol.

[42] Block AK, Mendoza J, Rowley A, Stuhl C, Meagher RL (2021). Approaches for assessing the impact of *Zea mays* (Poaceae) on the behavior of *Spodoptera frugiperda* (Lepidoptera: Noctuidae) and its parasitoid *Cotesia marginiventris* (Hymenoptera: Braconidae). Florida Entomol.

[43] Pinto Zevallos DM, Strapasson P, Zarbin PH (2016). Herbivore-induced volatile organic compounds emitted by maize: electrophysiological responses in *Spodoptera frugiperda* females. Phytochem Lett.

[44] Galizia CG, Rössler W (2010). Parallel olfactory systems in insects: anatomy and function. Annu Rev Entomol.

[45] Yan XZ, Deng CP, Sun X, Chi H (2014). Effects of various degrees of antennal ablation on mating and oviposition preferences of the diamondback moth, *Plutella xylostella*. J Integr Agric.

[46] Hansson B (1995). Olfaction in lepidoptera. Experientia.

[47] Cury KM, Prud’homme B, Gompel N (2019). A short guide to insect oviposition: when, where and how to lay an egg. J Neurogenet.

[48] Pophof B (2004). Pheromone-binding proteins contribute to the activation of olfactory receptor neurons in the silkmoths *Antheraea polyphemus* and *Bombyx mori*. Chem Senses.

[49] Laughlin JD, Ha TS, Jones DN, Smith DP (2008). Activation of pheromone-sensitive neurons is mediated by conformational activation of pheromone-binding protein. Cell.

[50] Kaissling KE (2009). Olfactory perireceptor and receptor events in moths: a kinetic model revised. J Comp Physiol A.

[51] Leal WS, Chen AM, Ishida Y, Chiang VP, Erickson ML, Morgan TI, Tsuruda JM (2005). Kinetics and molecular properties of pheromone binding and release. Proc Natl Acad Sci.

[52] Haverkamp A, Hansson BS, Knaden M (2018). Combinatorial codes and labeled lines: how insects use olfactory cues to find and judge food, mates, and oviposition sites in complex environments. Front Physiol.

[53] Dweck HK, Ebrahim SA, Kromann S, Bown D, Hillbur Y, Sachse S (2013). Olfactory preference for egg laying on citrus substrates in *Drosophila*. Curr Biol.

[54] Wang C, Li GN, Miao CJ, Zhao M, Wang B, Guo XR (2020). Nonanal modulates oviposition preference in female *Helicoverpa assulta* (Lepidoptera: Noctuidae) via the activation of peripheral neurons. Pest Manag Sci.

[55] Liu Y, Xiao HM, Mei Y, Yang Y, Ye XH, Chen AD (2019). Evolutionary analysis of chemoreception related gene families of *Spodoptera frugiperda*. J Environ Entomol.

[56] Gouin A, Bretaudeau A, Nam K, Gimenez S, Aury JM, Duvic B (2017). Two genomes of highly polyphagous lepidopteran pests (*Spodoptera frugiperda*, Noctuidae) with different host-plant ranges. Sci Rep.

[57] Guo JM, Liu XL, Liu SR, Wei ZQ, Han WK, Guo YZ (2020). Functional characterization of sex pheromone receptors in the fall armyworm (*Spodoptera frugiperda*). Insects.

[58] Honda K (1995). Chemical basis of differential oviposition by lepidopterous insects. Arch Insect Biochem Physiol.

[59] Renwick J, Chew F (1994). Oviposition behavior in Lepidoptera. Annu Rev Entomol.

[60] Rojas JC, Kolomiets MV, Bernal JS (2018). Nonsensical choices? Fall armyworm moths choose seemingly best or worst hosts for their larvae, but neonate larvae make their own choices. PLoS one.

[61] Carroll MJ, Schmelz EA, Meagher RL, Teal PE (2006). Attraction of *Spodoptera frugiperda* larvae to volatiles from herbivore-damaged maize seedlings. J Chem Ecol.

[62] Helms AM, De Moraes CM, Mescher MC, Tooker JF (2014). The volatile emission of *Eurosta solidaginis* primes herbivore-induced volatile production in *Solidago altissima* and does not directly deter insect feeding. BMC Plant Biol.

[63] Mescher MC, De Moraes CM (2015). Role of plant sensory perception in plant–animal interactions. J Exp Bot.

[64] Dai J, Deng J, Du J (2008). Development of bisexual attractants for diamondback moth, *Plutella xylostella* (Lepidoptera: Plutellidae) based on sex pheromone and host volatiles. Appl Entomol Zool.

[65] Reddy G, Guerrero A (2000). Behavioral Responses of the Diamondback Moth, *Plutella xylostella*, to green leaf volatiles of *Brassica oleracea* Subsp. *capitata*. J Agric Food Chem.

[66] Li P, Zhu J, Qin Y (2012). Enhanced attraction of *Plutella xylostella* (Lepidoptera: Plutellidae) to pheromone-baited traps with the addition of green leaf volatiles. J Econ Entomol.

[67] Sun YL, Dong JF, Song YQ, Wang SL (2021). GOBP1 from the Variegated Cutworm *Peridroma saucia* (Hübner)(Lepidoptera: Noctuidae) displays high binding affinities to the behavioral attractant (*Z*)-3-Hexenyl-acetate. Insects.

[68] Sans A, Moran M, Riba M, Guerrero A, Roig J, Gemeno C (2016). Plant volatiles challenge inhibition by structural analogs of the sex pheromone in Lobesia botrana (Lepidoptera: Tortricidae). Eur J Entomol.

[69] Allmann S, Späthe A, Bisch-Knaden S, Kallenbach M, Reinecke A (2013). Feeding-induced rearrangement of green leaf volatiles reduces moth oviposition. Elife.

[70] Tanaka K, Uda Y, Ono Y, Nakagawa T, Suwa M, Yamaoka R (2009). Highly selective tuning of a silkworm olfactory receptor to a key mulberry leaf volatile. Curr Biol.

[71] Zhang J, Liu C, Yan S, Liu Y, Guo M, Dong S (2013). An odorant receptor from the common cutworm (*Spodoptera litura*) exclusively tuned to the important plant volatile cis-3-Hexenyl acetate. Insect Mol Biol.

[72] Liu CC, Liu Y, Guo MB, Cao DP, Dong SL, Wang GR (2014). Narrow tuning of an odorant receptor to plant volatiles in *Spodoptera exigua* (Hübner). Insect Mol Biol.

[73] Zhang J, Wang B, Dong SL, Cao DP, Dong JF, Walker WB (2015). Antennal transcriptome analysis and comparison of chemosensory gene families in two closely related noctuidae moths, *Helicoverpa armigera* and *Helicoverpa assulta*. PLoS one.

[74] Cui WC, Wang B, Guo MB, Liu Y, Jacquin Joly E, Yan SC (2018). A receptor-neuron correlate for the detection of attractive plant volatiles in *Helicoverpa assulta* (Lepidoptera: Noctuidae). Insect Biochem Mol Biol.

[75] Ying WS, Zhan ZQ, Ting TY, Li MQ, Fei WR, Fang ZM (2019). Artificial diets and rearing technique of *Spodoptera frugiperda* (J.E. Smith) in laboratory. Environ Entomol.

[76] Zhang MM, Cui ZH, Zhang N, Xie GL, Wang WK, Chen L (2021). Electrophysiological and behavioral responses of *Holotrichia parallela* to volatiles from peanut. Insects.

[77] Chen L, Li YY, Shao KM (2019). A practical technique for electrophysiologically recording from lamellated antenna of scarab beetle. J Chem Ecol.

[78] Qiu L, He L, Tan XP, Zhang ZB, Wang Y, Li XW (2020). Identification and phylogenetics of *Spodoptera frugiperda* chemosensory proteins based on antennal transcriptome data. Comp Biochem Physiol D Genomics Proteomics.

[79] Pfaffl MW (2001). A new mathematical model for relative quantification in real-time RT–PCR. Nucleic Acids Res.

[80] Rodríguez de la Noval C, Rodríguez Cabrera L, Izquierdo L, Espinosa LA, Hernandez D (2019). Functional expression of a peritrophin A-like SfPER protein is required, for larval development in *Spodoptera frugiperda* (Lepidoptera: Noctuidae). Sci Rep.

[81] Wohlers P, Tjallingii W (1983). Electroantennogram responses of aphids to the alarm pheromone (*E*)-*β*-farnesene. Entomol Exp Appl.

[82] Xu T, Xu M, Lu YY, Zhang WQ, Sun JH, Zeng RS (2021). A trail pheromone mediates the mutualism between ants and aphids. Curr Biol.

