# Supplementary figures and images for: A green leaf volatile, (Z)-3-hexenyl-acetate, mediates differential oviposition by Spodoptera frugiperda on maize and rice
Source: BMC Biol. 2023 Jun 19;21:140. doi: 10.1186/s12915-023-01642-x (PMC10280969; doi:10.1186/s12915-023-01642-x)

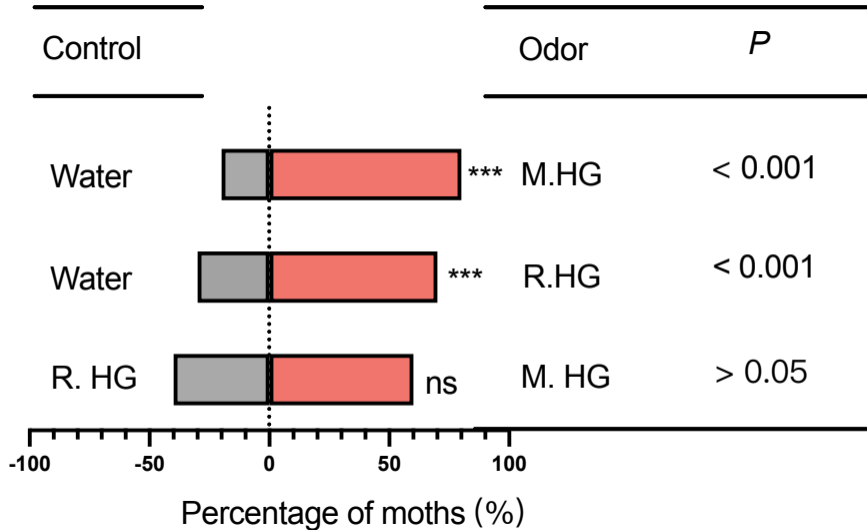

Supplement: Supplementary file 2 — Additional file 2: Figure S1. Olfactory preferences of female S. frugiperda to homogenates. Behavioral responses of female S. frugiperda to the maize homogenate vs. control, rice homogenate vs. control, and maize homogenate vs. rice homogenate (control: water, Chi-Square test, n = 30; ns, P > 0.05; *P < 0.05; ** P < 0.01; *** P < 0.001). [file 12915_2023_1642_MOESM2_ESM.pdf]

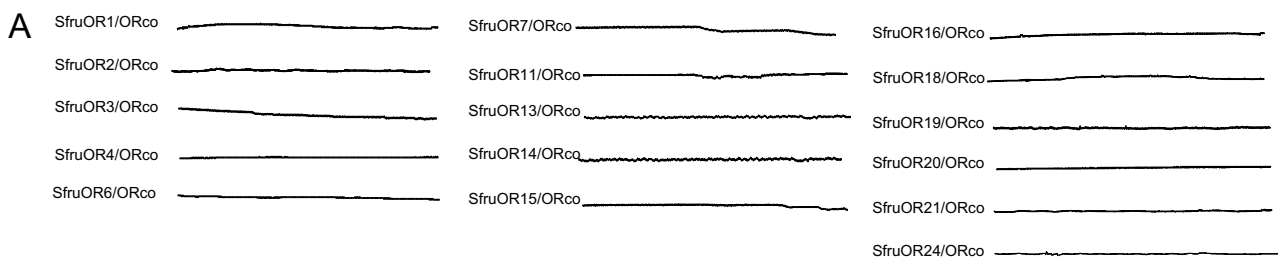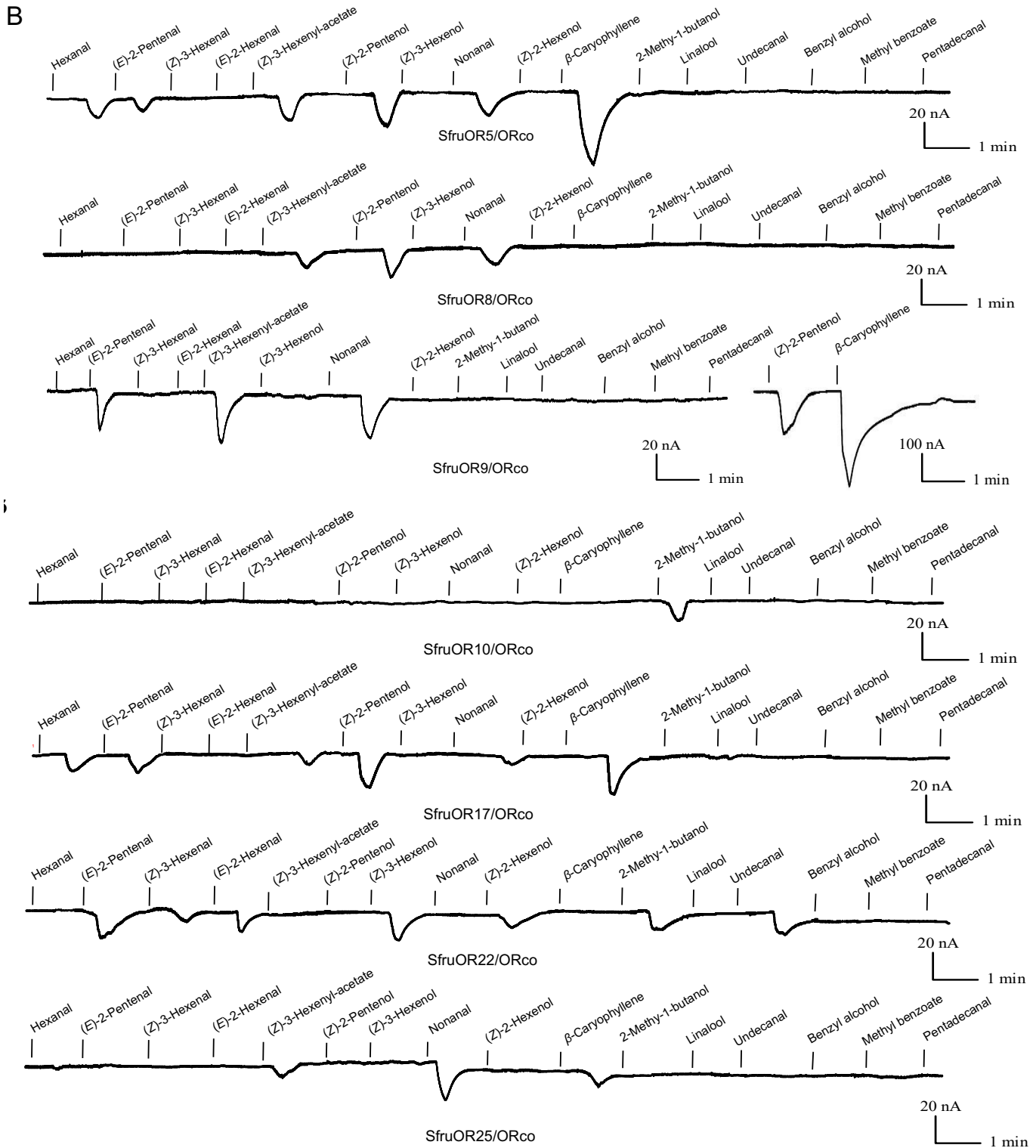

Supplement: Supplementary file 7 — Additional file 7: Figure S5. Two-electrode Voltage-clamp Recordings in Xenopus oocytes. A. None of the antenna ORs responded to the 16 VOCs (10-4 M). n = 6 (oocytes). B. Inward current responses of Xenopus oocytes expressing ORs to VOCs (10-4 M). n = 6 (oocytes). [file 12915_2023_1642_MOESM7_ESM.pdf]
